# Prevalence of potentially inappropriate prescribing in community-dwelling older adults: an application of STOPP/START version 3 to The Irish Longitudinal Study on Ageing (TILDA)

**DOI:** 10.1007/s41999-025-01201-3

**Published:** 2025-04-28

**Authors:** Ann Sinéad Doherty, Frank Moriarty, Fiona Boland, Barbara Clyne, Tom Fahey, Rose Anne Kenny, Denis O’Mahony, Emma Wallace

**Affiliations:** 1 Department of General Practice, School of Medicine, University College Cork, Western Gateway Building, Western Road, Cork, T12 XF62 Ireland; 2 School of Pharmacy and Biomolecular Sciences, RCSI University of Medicine and Health Sciences, Dublin, Ireland; 3 The Irish Longitudinal Study on Ageing (TILDA), Trinity College Dublin, Dublin, Ireland; 4 Data Science Centre, School of Population Health, RCSI University of Medicine and Health Sciences, Dublin, Ireland; 5 Department of Public Health and Epidemiology, School of Population Health, RCSI University of Medicine and Health Sciences, Dublin, Ireland; 6 Department of General Practice, RCSI University of Medicine and Health Sciences, Dublin, Ireland; 7 Department of Medical Gerontology, School of Medicine, Trinity College Dublin, Dublin, Ireland; 8 Department of Medicine, School of Medicine, University College Cork, Cork, Ireland; 9 Department of Geriatric and Stroke Medicine, Cork University Hospital, Cork, Ireland

**Keywords:** Potentially inappropriate prescribing, STOPP, START, Healthcare utilisation, Activities of daily living

## Abstract

**Purpose:**

Potentially inappropriate prescribing includes prescribing potentially inappropriate medicines (PIMs), where risk of medication-related harm may outweigh the clinical benefit(s), and potential prescribing omissions (PPOs), whereby clinically indicated medications are unprescribed without good reason. This study aimed to assess prevalence of PIMs and PPOs (subset of STOPP/START version 3) in older community-dwelling adults and any association with healthcare utilisation and functional decline over time.

**Methods:**

Retrospective cohort study of a nationally representative longitudinal study of ageing in Ireland (*n* = 3619) (2016–2018). Logistic regressions examined association of patient characteristics with PIMs/PPOs and between prevalent PIMs/PPOs and functional decline. Negative binomial regressions examined association between PIM/PPO with healthcare utilisation over time.

**Results:**

Participants’ mean age was 74.2 years (SD 6.99), 53.9% were female and were prescribed a mean of 4.02 (SD 3.16) medications. A total of 1123 (31.0%) participants experienced STOPP PIMs and 1309 (36.2%) START PPOs. STOPP PIMs were associated with increased hospital admissions (adjusted incident rate ratio (aIRR) 1.38, 95% confidence interval (CI) 1.08, 1.75), and functional decline (adjusted odds ratio (aOR) 1.46, 95% CI 1.11, 1.91) at follow-up. Age ≥ 75 years (aOR 1.32, 95% CI 1.10, 1.57) and three or more chronic conditions (aOR 5.19, 95% CI 3.69, 7.31) were significantly associated with START PPOs.

**Conclusion:**

Approximately one-third of study participants experienced STOPP PIMs, associated with an increased risk of hospital admissions and functional decline. START PPOs also occurred in over one-third, associated with increasing age and degree of multimorbidity. Balancing the risk: benefit of medications for older people with multimorbidity remains challenging.

**Supplementary Information:**

The online version contains supplementary material available at 10.1007/s41999-025-01201-3.

## Key summary points

**Aim:**

To assess the prevalence of the latest version of STOPP/START criteria (Version 3) in a nationally representative sample of older community-dwelling adults and explore any association with healthcare utilisation and functional decline over time.

**Findings:**

In a sample of 3619 older Irish adults, a total of 31% (*n* = 1123) experienced STOPP potentially inappropriate medications (PIMs) and exposure to any STOPP PIM was associated with increased hospital admissions and functional decline over time. Just over one-third of participants (36.2%; *n* = 1309) experienced START potential prescribing omissions (PPOs), with increasing age and number of chronic conditions associated with START PPOs.

**Message:**

Over one-third of participants experienced a subset of STOPP PIMs, another one-third experienced a subset of START PPOs. Balancing the relative benefits and risks of medication in older adults with multimorbidity continues to present challenges for both prescribers and older adults.

**Supplementary Information:**

The online version contains supplementary material available at 10.1007/s41999-025-01201-3.

## Introduction

Potentially inappropriate medications (PIMs) are medications where the potential for medication-related harm may outweigh the clinical benefit(s) to the patient. Potentially inappropriate prescribing (PIP) includes PIMs as well as potential prescribing omissions (PPOs), whereby clinically indicated medications are not prescribed [1].

Older adults are particularly vulnerable to PIP as they are more likely to live with multiple long-term health conditions. More than half of the global population aged ≥ 60 years are estimated to have multimorbidity, defined as the presence of two or more chronic conditions [2]. Consequently, older adults are more likely to experience polypharmacy (often defined as the use of five or more daily long-term prescribed medications), with international prevalence rates of 45%, and higher rates in frail older adults (59%) [3]. Age-related changes in physiology (e.g. renal function, liver function, body mass etc.) mean that often both the metabolism of and response to medication is altered in older adults, resulting in an underlying biological vulnerability to adverse drug reactions (ADRs) [4]. The prevalence of ADRs is higher amongst older adults (≥ 65 years), with more than one-quarter shown to experience an ADR and 1 in 10 hospital admissions associated with serious ADRs [5–7]. PIP is also associated with a range of other adverse outcomes including falls and functional decline [8]. Nevertheless, comparatively fewer studies have explored associations between PIP and functional decline, with limited exploration of the relationship for PPOs specifically [8]. Direct healthcare costs for those patients exposed to PIP are more than twice the total cost of those without PIP [9].

Explicit criteria to identify PIP are lists of medications or medication/disease combinations considered harmful when used in older adults. Numerous PIP explicit criteria for older adults have been published internationally including the US Beers’ list, French Laroche list and Norwegian General Practice (NORGEP) criteria [10–12]. In addition, the European Screening Tool of Older Person’s potentially inappropriate Prescriptions (STOPP) and the Screening Tool to Alert to Right Treatment (START) criteria assess PIMs and PPOs, respectively [13, 14]. Originally developed by expert consensus in Ireland, the 2008 STOPP/START criteria have undergone considerable revision since the first iteration. STOPP/START Version 3, published in 2023 represents an approximate 200% increase in the original number of prescribing indicators in version 1, reflecting Europe-wide consensus on newer medications and the expanding pharmacotherapy evidence base for older people [15].

Longitudinal cohort studies of ageing allow examination of temporal changes in PIP through the application of explicit criteria such as STOPP/START. In Ireland, The Irish Longitudinal Study on Ageing (TILDA), has recently been designated as the World Health Organisation Collaborating Centre for Longitudinal Studies on Ageing and the Life Course [16, 17]. Previous application of STOPP/START version 1 criteria to TILDA self-reported medication data identified prevalence rates of PIMs and PPOs of 14.6% and 30%, respectively [18]. When later examined in a subset of TILDA participants with linked administrative pharmacy claims data, the overall prevalence was higher; 57% and 41.8% for PIM and PPO, respectively [19]. This observable increase likely reflects the application of more indicators due to additional data provided by the linked pharmacy claims data. The aim of this study was to examine the prevalence of PIM and PPO, identified by STOPP/START Version 3, in older Irish adults and to examine any association with healthcare utilisation and functional decline over time.

## Methods

The Strengthening the Reporting of Observational Studies in Epidemiology (STROBE) statement has been used in reporting this study (see Additional File 1) [20].

### Study design, population and data collection

A retrospective cohort study was conducted using a nationally representative longitudinal study of ageing in Ireland (TILDA). TILDA respondents are community-dwelling adults aged ≥ 50 years. Baseline data collection was conducted between 2009 and 2011, with respondents followed up every two years thereafter. Data collection procedures have been detailed elsewhere [16, 17], but in summary involved the completion of a computer-assisted personal interview (CAPI) and self-completion questionnaire (SCQ) at each wave, that includes questions regarding current medications and diagnoses. Participants complete the CAPI and SCQ at each wave (every 2 years) and the health assessment at alternate waves (e.g. Waves 1 and 3). Medication data, collected by CAPI, demonstrates both internal and external validity [21]. Morbidity self-reports from TILDA participants have shown moderate to very good agreement with medication-based morbidity assessments for many chronic conditions included in the present study [22]. This study utilised data from the CAPI and SCQ for waves 1–5. For this study, Wave 4 (2016) was used as the baseline assessment point with demographical characteristics at Wave 4 used to describe the sample. Follow-up outcomes (healthcare utilisation and functional decline) were examined at Wave 5 (2018). Data from Waves 1–3 were examined for some indicators where prior use of a medication or reporting of a disease was relevant e.g. primary prevention of cardiovascular disease required an examination as to whether cardiovascular disease was ever reported at an earlier wave. At Wave 4, a total of 5973 respondents completed data collection. Data pertaining to those who were aged ≥ 65 years at Wave 4 (*N* = 3619) were included in the analysis.

### Variables

#### Primary outcome variables

The primary outcome of interest was exposure to PIMs, assessed using STOPP criteria, and PPOs, assessed using START criteria (Version 3) at Wave 4. A subset of STOPP/START Version 3 were applied in line with previous applications of Version 1 to earlier TILDA data [18, 19]. The selected subset of indicators was based on the availability of medication and disease information within the database. Sixty STOPP criteria (45% of 133 STOPP criteria) and 16 START criteria (28% of 57 START criteria) were applied. In the absence of disease information, prescription medications for the treatment of certain conditions were used as proxies e.g. the use of allopurinol or colchicine was used as a marker of history of gout. A detailed description of how the various indicators were defined is provided in Additional File 2.

#### Secondary outcome variables

Healthcare utilization (self-reported use over the previous 12 months) at Wave 5 (2018) was operationalized as the number of general practitioner (GP) visits; Emergency Department (ED) visits; number of outpatient visits; and number of hospital admissions. Self-reported limitations in activities of daily living (ADLs) and instrumental activities of daily living (IADLs) were reported during the CAPI. This is measured in TILDA as limitations in ADLs (help with walking across a room, dressing, bathing, eating, getting in and out of bed, and using the toilet) and IADLs (preparing meals, shopping for groceries, making telephone calls, taking medications and managing money) [23]. The total number of both ADL and IADL limitations was calculated at waves 4 and 5. To operationalise functional decline, a binary variable was computed to capture those participants who acquired at least one additional ADL or IADL impairment between waves 4 and 5.

#### Explanatory variables

Explanatory variables included age group (65–74 years; ≥ 75 years) and sex (male/female). To examine socioeconomic status three variables were included, i) educational attainment; ii) employment status; and iii) type of healthcare coverage. Educational attainment was categorised as primary level/none; secondary level; third level/higher education. Employment status was categorised as employed; retired; or other. Healthcare coverage was categorised as no cover; public General Medical Services (GMS) scheme cover only; private health insurance cover only; and dual GMS and private coverage. The GMS scheme provides access to free healthcare, as well as access to eligible prescriptions which are available via a low co-payment that is capped monthly per household. GMS scheme eligibility is based upon income-related means testing, with a higher income threshold set for those ≥ 70 years. The scheme provides coverage for more than 60% of those aged ≥ 65 years and approximately 80% of those aged ≥ 75 years in Ireland [24].

Multimorbidity was defined as the number of chronic conditions, from a list of 21 conditions captured during data collection and previously applied by Larkin et al. [25]. Further detail regarding the list of conditions included is reported in Additional File 3. Multimorbidity was further categorised as none, one, two, or ≥ 3 chronic conditions, in line with previous TILDA studies [18, 19]. Polypharmacy was categorised as 0–4 medications (no polypharmacy); 5–9 medications (polypharmacy); ≥ 10 medications (hyperpolypharmacy), based upon self-reported regular medication use reported during the CAPI. Participants were asked to report all medications taken regularly, including prescription and non-prescription medication, over-the-counter medications and supplements.

### Analytical plan

Descriptive statistics were used to summarise the population. The overall prevalence of PIM/PPO (any STOPP/START indicator) and the prevalence per individual criteria were calculated as the proportion of all eligible participants aged ≥ 65 years. The prevalence of PIM/PPO was also calculated as the proportion of overall disease or medication prevalence (e.g. drugs with potent anticholinergic/antimuscarinic effects in patients with dementia as a proportion of dementia prevalence). Further detail on the drug/disease denominator used for each criterion is provided in Additional File 2.

Univariate associations between age, sex, education, employment status, healthcare coverage, multimorbidity, and polypharmacy and presence of PIM/PPO (with none as referent) were explored using logistic regression analyses, reported as odds ratios (ORs) with 95% confidence intervals (CI). Multivariable associations were explored using multivariable logistic regression analysis.

Separate negative binomial regressions were conducted to examine the relationship between experiencing PIM/PPO (any PIM/PPO (model 1) and 0, 1 and ≥ 2 STOPP/START criteria (model 2)) at Wave 4 and healthcare utilisation at Wave 5. The models were adjusted for relevant confounders (age, sex, education, employment status, healthcare coverage, multimorbidity, polypharmacy, and healthcare utilisation at Wave 4). Results are reported as incident rate ratios (IRRs) with 95% CIs. Logistic regression analyses examined the relationship between experiencing PIM/PPO and functional decline from Wave 4 to Wave 5, adjusted for relevant confounders for each outcome (age, sex, education, employment status, healthcare coverage, multimorbidity, polypharmacy, and the presence of any ADL/IADL impairment at Wave 4). Results are reported as odds ratios with 95% CIs. Data analyses were performed using Stata version 18 (StataCorp) with an alpha level of *p* < 0.05.

## Results

Descriptive statistics for the overall sample (*n* = 3619) and by experience of any PIM and any PPO are presented in Table 1. The mean age of study participants was 74.20 years (SD 6.99) and just over half (53.94%) were female. The mean number of regular medications was 4.02 (SD 3.16). Approximately two-fifths of participants were prescribed five or more medications, with 6.02% of participants prescribed ≥ 10 medications.

**Table 1:** Descriptive statistics for study participants

	Overall *N* = 3619 *n* (%)	No STOPP *N* = 2496 *n* (%)	Any STOPP *N* = 1123 *n* (%)	No START *N* = 2310 *n* (%)	Any START *N* = 1309 *n* (%)
Age in years, *M* (SD)	74.20 (6.99)	73.67 (6.88)	75.37 (7.09)	73.68 (6.89)	75.12 (7.06)
65–74 years	2114 (58.41)	1532 (61.38)	582 (51.83)	1423 (61.60)	691 (52.79)
≥ 75 years	1505 (41.59)	964 (38.62)	541 (48.17)	887 (38.40)	618 (47.21)
Sex					
Male	1667 (46.06)	1191 (47.72)	476 (42.39)	1165 (50.43)	502 (38.35)
Female	1952 (53.94)	1305 (52.28)	647 (57.61)	1145 (49.57)	807 (61.65)
Education					
Primary/none	1194 (32.99)	763 (30.57)	431 (38.38)	761 (32.94)	433 (33.08)
Secondary	1316 (36.36)	919 (36.82)	397 (35.35)	818 (35.41)	498 (38.04)
Third/higher	1109 (30.64)	814 (32.61)	295 (26.27)	731 (31.65)	378 (28.88)
Employment status^a^					
Employed	456 (12.61)	360 (14.43)	96 (8.55)	354 (15.34)	102 (7.79)
Retired	2559 (70.75)	1741 (69.81)	818 (72.84)	1610 (69.76)	949 (72.50)
Other	602 (16.64)	393 (15.76)	209 (18.61)	344 (14.90)	258 (19.71)
Health insurance^b^					
Not covered	122 (4.04)	100 (4.82)	22 (2.34)	87 (4.50)	35 (3.23)
Private insurance only	734 (24.32)	585 (28.18)	149 (15.82)	518 (26.80)	216 (19.91)
GMS cover only	1319 (43.70)	838 (40.37)	481 (51.06)	804 (41.59)	515 (47.47)
Dual cover	843 (27.93)	553 (26.64)	290 (30.79)	524 (27.11)	319 (29.40)
Number of morbidities, *M *(SD)	2.36 (1.62)	2.09 (1.53)	2.97 (1.66)	2.00 (1.48)	2.98 (1.67)
Multimorbidity category					
None	419 (11.58)	367 (14.70)	52 (4.63)	356 (15.41)	63 (4.81)
1 chronic condition	738 (20.39)	578 (23.16)	160 (14.25)	560 (24.24)	178 (13.60)
2 chronic conditions	940 (25.97)	693 (27.76)	247 (21.99)	628 (27.19)	312 (23.83)
≥ 3 chronic conditions	1522 (42.06)	858 (34.38)	664 (59.13)	766 (33.16)	756 (57.75)
Number of prescribed medications, *M* (SD)	4.02 (3.16)	3.17 (2.73)	6.09 (3.18)	3.65 (3.06)	4.69 (3.21)
Polypharmacy category					
0–4 medications	2213 (61.15)	1822 (73.00)	391 (34.82)	1523 (65.93)	690 (52.71)
5–9 medications	1188 (32.83)	614 (24.60)	574 (51.11)	679 (29.39)	509 (38.88)
≥ 10 medications	218 (6.02)	60 (2.40)	158 (14.07)	108 (4.68)	110 (8.40)

^a^Data missing for two participants; ^b^ data missing for 601 participants

Just under one-third of participants (*n* = 1123, 31.03%) experienced one or more PIMs, with 814 participants (22.49%) having one STOPP PIM indicator and 309 (8.54%) with two or more STOPP PIM indicators (Table 2). Of the total study population (*n* = 3619) the most prevalent STOPP indicator was the use of aspirin for primary prevention of cardiovascular disease (14.78%). Across 15 different drug classes a total of 125 participants (3.45%) reported duplicate prescribing (Table 2). Other prevalent indicators included the use of NSAIDs and z-drugs beyond their recommended duration, representing 2.76% and 2.57%, respectively. When both indicators were expressed as the proportion of drug class prescribing the prevalence was found to be 68.03% and 59.62%, respectively. A total of 45.45% of TILDA participants aged ≥ 85 years with established frailty were found to be using statins for primary cardiovascular prevention.

**Table 2:** STOPP Version 3 criteria applied to TILDA data for study participants aged ≥ 65 years (*N* = 3619)

STOPP criteria description	PIM (n)	PIM (%)	Prescribing per indication (%)^a^
Indication of medication			
Any drug prescribed beyond the recommended duration			
Z-drugs	93	2.57	59.62
NSAIDs	100	2.76	68.03
Any duplicate drug class prescription	125	3.45	4.73
Duplicate NSAIDs	11	0.3	4.82
Duplicate SSRIs	7	0.19	3.52
Duplicate loop diuretics	4	0.11	2
Duplicate ACEIs	12	0.33	1.88
Duplicate anticoagulants	6	0.17	1.81
Duplicate antipsychotics	6	0.17	8.45
Duplicate opioids	11	0.3	6.04
Duplicate stimulant laxatives	0	–	–
Duplicate thiazides	0	–	–
Duplicate beta blockers	16	0.44	1.94
Duplicate ARBs	14	0.39	2.01
Duplicate CCBs	9	0.25	1.44
Duplicate statins	25	0.69	1.59
Duplicate benzodiazepines	8	0.22	5.63
Duplicate TCAs	0	–	–
Cardiovascular system			
Beta-blocker in combination with verapamil or diltiazem	11	0.3	17.19
Beta-blocker as monotherapy for uncomplicated hypertension	42	1.16	4.12
Loop diuretic as first-line treatment for hypertension unless there is concurrent heart failure	24	0.66	1.64
Thiazide diuretic with a history of gout (defined as prior use of anti-gout medication)	4	0.11	2.53
Loop diuretic for treatment of hypertension with concurrent urinary incontinence	21	0.58	6.46
Centrally acting antihypertensives	0	–	–
Phosphodiesterase type-5 inhibitors with concurrent nitrate therapy for angina	1	0.03	1.3
Statins for primary cardiovascular prevention in persons aged ≥ 85 and established frailty	10	0.28	45.45
Long-term systemic i.e. non-topical NSAIDs with known history of coronary, cerebral or peripheral vascular disease	14	0.39	1.43
Long-term antipsychotics with known history of coronary, cerebral or peripheral vascular disease	11	0.30	1.12
NSAIDs or systemic corticosteroids with heart failure requiring loop diuretic therapy	1	0.03	7.69
Coagulation system			
Ticlopidine in any circumstances	0	–	–
Antiplatelet agents as alternatives to vitamin K antagonists, direct thrombin inhibitors or factor Xa inhibitors for stroke prevention in patients with chronic atrial fibrillation	30	0.83	19.48
NSAIDs and vitamin K antagonist, direct thrombin inhibitor or factor Xa inhibitors in combination	6	0.17	2.48
Direct thrombin inhibitor (e.g. dabigatran) and diltiazem or verapamil	7	0.19	10.94
Apixaban, dabigatran, edoxaban, rivaroxaban and P-glycoprotein (P-gp) drug efflux pump inhibitors	15	0.41	11.72
Aspirin for primary prevention in cardiovascular disease	535	14.78	21.80
Central nervous system			
Tricyclic antidepressants (TCAs) in patients with dementia, glaucoma or recent falls	35	0.97	3.49
Benzodiazepines for ≥ 4 weeks	64	1.77	55.65
Benzodiazepines for agitated behaviour or psychotic symptoms of dementia	0	–	–
Benzodiazepines for insomnia for ≥ 2 weeks	27	0.75	23.48
Z-drugs (zolpidem, zopiclone, zaleplon) for insomnia for ≥ 2 weeks	39	1.08	25.00
Antipsychotics (i.e. other than clozapine or quetiapine) in those with parkinsonism	1	0.03	2.5
Anticholinergic/antimuscarinic drugs to treat extra-pyramidal side-effects of antipsychotic medications	0	–	–
Drugs with potent anticholinergics/antimuscarinic effects in patients with dementia	7	0.19	8.14
Antipsychotics as hypnotics, unless sleep disorder is due to psychosis or BPSD effects of dementia	10	0.28	8.4
Acetylcholinesterase inhibitors with concurrent treatment with drugs that induce persistent bradycardia such as beta-blockers, digoxin, diltiazem, verapamil	15	0.41	1.68
Memantine with known current or previous seizure disorder	0	–	–
Nootropics in dementia	0	–	–
Levodopa or dopamine agonists for treatment of extra-pyramidal side-effects of antipsychotics (inappropriate prescribing cascade)	0	–	–
Gastrointestinal system			
Prochlorperazine or metoclopramide with Parkinsonism	0	–	–
Corticosteroids with a history of peptic ulcer disease	2	0.06	0.62
Respiratory system			
Theophylline as monotherapy for COPD	0	–	–
Systemic corticosteroids instead of inhaled corticosteroids for maintenance therapy in moderate-severe COPD	5	0.14	9.43
Long-acting muscarinic antagonists with a history of glaucoma	4	0.11	3.05
Musculoskeletal system			
Non-steroidal anti-inflammatory drugs (NSAIDs) other than COX-2 selective agents with history of peptic ulcer disease or gastrointestinal bleeding, unless with concurrent PPI or H2 antagonist	14	0.39	4.32
Long-term corticosteroids (> 3 months) as monotherapy for rheumatoid arthritis	4	0.11	1.30
Corticosteroids (other than periodic intra-articular injections for mono-articular pain) for osteoarthritis	20	0.55	2.77
NSAID with concurrent corticosteroids for treatment of arthritis/rheumatism of any kind	3	0.08	0.22
Oral bisphosphonates in patients with a current or recent history of upper gastrointestinal	18	0.5	5.56
Long-term opioids for osteoarthritis	32	0.88	6.13
Urogenital system			
Systemic antimuscarinic drugs with dementia or chronic cognitive impairment	8	0.22	9.3
Systemic antimuscarinic drugs with glaucoma	11	0.30	8.4
Endocrine system			
Sulphonylureas with a long half-life with type 2 diabetes mellitus	0	–	–
Thiazolidenediones with heart failure	0	–	–
Systemic oestrogens with a history of breast cancer	1	0.03	0.89
Vasopressin analogues for urinary incontinence or urinary frequency	1	0.03	0.14
Drug classes that predictably increase falls risk			
Benzodiazepines in patients with recurrent falls	18	0.50	4.83
Antipsychotic drugs in patients with recurrent falls	10	0.28	2.68
Hypnotic Z-drugs in patients with recurrent falls	30	0.83	8.04
Anti-epileptic drugs in patients with recurrent falls	47	1.30	12.60
First generation antihistamines in patients with recurrent falls	2	0.06	0.54
Opioids in patients with recurrent falls	39	1.08	10.46
Antidepressants in patients with recurrent falls	97	2.68	26.01
Centrally acting antihypertensives	0	–	–
Antimuscarinics for treatment of overactive bladder or urge incontinence	44	1.22	6.33
Analgesic drugs			
Topical lidocaine patch for treatment of chronic osteoarthritis pain	13	0.36	1.54
Antimuscarinic/anticholinergic drug burden			
Concomitant use of two or more drugs with antimuscarinic/anticholinergic properties	19	0.53	8.15
Any STOPP indicator	1123	31.03	
1 indicator	814	22.49	–
≥ 2 indicators	309	8.54	–

^a^ Calculated as the proportion of overall disease or medication prevalence (e.g. use of medication with potent anticholinergic/antimuscarinic effects in participants with dementia as a proportion of dementia prevalence). Further detail on the disease/medication denominator used for each criterion is provided in Additional File 2

Just over one-third of participants (36.17%, *n* = 1309) experienced one or more PPOs, with 788 (21.77%) with one START indicator and 521 (14.40%) with two or more START indicators (Additional File 4). The most prevalent START indicator was the absence of an angiotensin converting enzyme inhibitor (ACEI) in those with coronary artery disease (*n* = 601; 16.61%), which represented 74.11% of the prescribing per indication. Other prevalent START indicators include the absence of vitamin D in patients with known osteoporosis (*n* = 439; 12.13%), no statin therapy in those a documented history of coronary, cerebral or peripheral vascular disease and no established frailty (*n* = 341; 9.42%), no antiplatelet therapy in those with a documented history of coronary, cerebral or peripheral vascular disease (7.79%), and the absence of a non-tricyclic antidepressant in those who met the Center for Epidemiologic Studies Depression 8-item scale (CESD-8) threshold for depression (7.38%) (Additional File 4, Table S5).

Of the 3619 TILDA participants aged ≥ 65 years at Wave 4, 449 (12.41%) had both STOPP and START indicators. A total of 674 (18.62%) had a STOPP indicator only and a further 860 (23.76%) had a START indicator only. Approximately two-fifths of the sample (45.21%, *n* = 1636) did not have either a STOPP or START criterion.

Unadjusted and adjusted associations between participant characteristics and experience of any STOPP and any START indicators are presented in Table 3. In the adjusted model, significant associations for any STOPP indicator were observed for those aged ≥ 75 years (adjusted (a) OR 1.43, 95% CI 1.25, 1.64), females (aOR 1.21, 95% CI 1.00, 1.46), those with one or more chronic conditions (Table 3), and for those with 5–9 daily medications (aOR 3.70, 95% CI 3.05, 4.47) and 10 or more daily medications (aOR 9.03, 95% CI 6.27, 13.00). Those with university or higher level educational attainment were less likely to receive any STOPP indicator (aOR 0.78, 95% CI 0.61, 0.99).

**Table 3:** Unadjusted (*n* = 3619) and adjusted (*n* = 3016) logistic regression for study participants with at least one STOPP *or* at least one START indicator

Characteristic	Any STOPP Unadjusted			Any STOPP Adjusted			Any START Unadjusted			Any START Adjusted		
	OR	95%CI	*p*	OR	95%CI	*p*	OR	95%CI	*p*	OR	95%CI	*p*
Age ≥ 75 years^a^	**1.48**	**1.28, 1.70**	**< 0.001**^**h**^	0.97	0.80, 1.17	0.728	**1.43**	**1.25, 1.64**	**< 0.001**	**1.32**	**1.10, 1.57**	**0.002**
Female sex^b^	**1.24**	**1.08, 1.43**	**0.003**	**1.21**	**1.00, 1.46**	**0.048**	**1.64**	**1.42, 1.88**	**< 0.001**	**1.30**	**1.09, 1.55**	**0.003**
Education^c^												
Secondary level	**0.76**	**0.65, 0.90**	**0.002**	0.84	0.68, 1.03	0.091	1.07	0.91, 1.26	0.414	1.17	0.97, 1.42	0.105
Third/higher level	**0.64**	**0.54, 0.77**	**< 0.001**	**0.78**	**0.61, 0.99**	**0.043**	0.91	0.77, 1.08	0.274	1.09	0.87, 1.37	0.447
Employment^d^												
Retired	**1.76**	**1.39, 2.24**	**< 0.001**	0.96	0.71, 1.30	0.793	**2.04**	**1.62, 2.59**	**< 0.001**	**1.89**	**1.41, 2.54**	**< 0.001**
Other	**1.99**	**1.51, 2.64**	**< 0.001**	0.98	0.69, 1.41	0.932	**2.60**	**1.98, 3.42**	**< 0.001**	**1.98**	**1.40, 2.80**	**< 0.001**
Health insurance^e^												
Private insurance only	1.16	0.70, 1.90	0.562	1.19	0.69, 2.03	0.527	1.04	0.68, 1.58	0.868	0.85	0.54, 1.34	0.490
GMS cover only	**2.61**	**1.62, 4.19**	**< 0.001**	1.38	0.81, 2.34	0.237	**1.59**	**1.06, 2.39**	**0.025**	0.79	0.50, 1.25	0.324
Dual cover	**2.38**	**1.47, 3.86**	**< 0.001**	1.48	0.86, 2.53	0.156	1.51	0.99, 2.29	0.051	0.75	0.47, 1.18	0.213
Multimorbidity^f^												
1 condition	**1.95**	**1.39, 2.74**	**< 0.001**	**1.62**	**1.10, 2.39**	**0.014**	**1.90**	**1.31, 2.46**	**< 0.001**	**1.83**	**1.28, 2.63**	**0.001**
2 conditions	**2.51**	**1.82, 3.48**	**< 0.001**	**1.65**	**1.13, 2.40**	**0.009**	**2.81**	**2.08, 3.79**	**< 0.001**	**2.76**	**1.96, 3.89**	**< 0.001**
≥ 3 conditions	**5.46**	**4.02, 7.43**	**< 0.001**	**2.40**	**1.66, 3.48**	**< 0.001**	**5.58**	**1.49, 7.42**	**< 0.001**	**5.19**	**3.69, 7.31**	**< 0.001**
Polypharmacy^g^												
5–9 medications	**4.36**	**3.72, 5.10**	**< 0.001**	**3.70**	**3.05, 4.47**	**< 0.001**	**1.65**	**1.13, 1.91**	**< 0.001**	1.08	0.90, 1.30	0.390
≥ 10 medications	**12.27**	**8.94, 16.84**	**< 0.001**	**9.03**	**6.27, 13.00**	**< 0.001**	**2.25**	**1.70, 2.97**	**< 0.001**	1.32	0.95, 1.84	0.098

^a^Referent is 65–74 years

^b^Referent is male sex

^c^Referent is primary level/none

^d^Referent is employed; missing data for two participants

^e^Referent is no cover; missing data for 601 participants

^f^Referent is no chronic conditions

^g^Referent is 0–4 regular medications

^h^ Associations with p <0.05 are in bold

When likelihood for any START indicator was examined in the adjusted model (Table 3), significant associations were identified for those aged ≥ 75 years (aOR 1.32, 95% CI 1.10, 1.57), females (aOR 1.30, 95% CI 1.09, 1.55), those who were retired (aOR 1.89, 95% CI 1.41, 2.54), or in an employment status other than employed (aOR1.98, 95% CI 1.40, 2.80), and those with one or more chronic conditions (Table 3). Compared to those with no chronic conditions, those with three or more chronic conditions were more than five times more likely to experience a START criterion PPO (aOR 5.19, 95% CI 3.69, 7.31).

In the 12 months prior to the Wave 5 TILDA interview, the average number of GP visits was 4.18 (SD 3.87, *N* = 2991). A total of 2.19% experienced one or more ED visits (*M* = 0.30, SD = 0.74, *N* = 3006) and 47.58% experienced one or more outpatient visits (*M* = 1.81, SD = 6.45, *N* = 2999). Approximately one fifth of the sample (19.65%) experienced one or more hospital admissions (M = 0.35, SD = 1.31, *N* = 3007). In total, 434 (14.41%, *N* = 3012) participants manifested functional decline between Wave 4 and Wave 5. Results of the secondary outcome analysis are summarised in Tables 4 and 5. In the adjusted models, significant associations were observed between any STOPP indicator at Wave 4 and the number of hospital admissions in the 12 months prior to the Wave 5 interview (adjusted IRR 1.38, 95% CI 1.08, 1.75; Table 4, model 1), as well as functional decline between Wave 4 and Wave 5 (aOR 1.46, 95% CI 1.11, 1.91; Table 5, model 1). When examined as a categorical exposure, those with two or more STOPP criteria had significantly more hospital admissions (Table 4, model 2) and were more likely to experience functional decline (Table 5, model 2). In contrast, those who experienced any START indicator had more GP visits (adjusted IRR 1.07, 95% CI 1.01, 1.13; Table 4, model 1), more ED visits (adjusted IRR 1.23, 95% CI 1.03, 1.48; Table 4, model 1) and more outpatient visits (adjusted IRR 1.21, 95% CI 1.02, 1.42; Table 4, model 1), but no significant associations were observed for hospital admissions nor functional decline.

**Table 4:** Association between STOPP/START and healthcare utilization outcomes using negative binomial regression (incident rate ratios (IRR), 95% confidence intervals (CIs))

	*M* (SD)^a^	GP visits (*n* = 2487) Adjusted IRR (95% CI)^b^	
		Model 1^c^	Model 2^d^
Any STOPP^e^ (*n* = 909)	4.98 (4.79)	1.06 (1.00, 1.13), *p* = 0.051	–
Number of STOPP PIMS			
0 STOPP (*n* = 2082)	3.83 (3.33)	–	(Referent)
1 STOPP (*n* = 658)	4.69 (4.28)	–	1.05 (1.00, 1.13), *p* = 0.064
≥ 2 STOPP (*n* = 251)	5.72 (5.87)	–	1.06 (0.96, 1.17), *p* = 0.267
Any START^e^ (*n* = 1062)	2.09 (6.77)	**1.07 (1.01, 1.13)**, ***p* = 0.018**^**f**^	–
Number of START PPOs			
0 START (*n* = 1933)	3.89 (3.57)	–	(Referent)
1 START (*n* = 651)	4.39 (3.49)	–	**1.07 (1.00, 1.15)**, ***p* = 0.038**
≥ 2 START (*n* = 407)	5.19 (5.35)	–	1.07 (0.99, 1.16), *p* = 0.097

	*M* (SD)^a^	ED visit (*n* = 2500) Adjusted IRR (95% CI)^b^	
		Model 1^c^	Model 2^d^
Any STOPP^e^ (*n* = 915)	0.37 (0.85)	0.91 (0.74, 1.11), *p* = 0.335	–
Number of STOPP PIMS			
0 STOPP (*n* = 2091)	0.27 (0.69)	–	(Referent)
1 STOPP (*n* = 662)	0.33 (0.81)	–	0.88 (0.71, 1.10), *p* = 0.260
≥ 2 STOPP (*n* = 253)	0.46 (0.93)	–	0.97 (0.72, 1.31), *p* = 0.847
Any START^e^ (*n* = 1066)	0.39 (0.80)	**1.23 (1.03, 1.48)**, ***p* = 0.025**	–
Number of START PPOs			
0 START (*n* = 1940)	0.22 (0.70)	–	(Referent)
1 START (*n* = 653)	0.36 (0.74)	–	1.20 (0.97, 1.49), *p* = 0.089
≥ 2 START (*n* = 413)	0.43 (0.87)	–	1.28 (1.00, 1.63), *p* = 0.050

	*M* (SD)^a^	Outpatient visit (*n* = 2493) Adjusted IRR (95% CI)^b^	
		Model 1^c^	Model 2^d^
Any STOPP^e^ (*n* = 914)	2.34 (8.50)	1.09 (0.92, 1.29), *p* = 0.304	–
Number of STOPP PIMS			
0 STOPP (*n* = 2085)	1.58 (5.30)	–	(Referent)
1 STOPP (*n* = 660)	2.21 (6.14)	–	1.08 (0.90, 1.30), *p* = 0.381
≥ 2 STOPP (*n* = 254)	2.67 (12.74)	–	1.11 (0.84, 1.46), *p* = 0.452
Any START^e^ (*n* = 1062)	2.09 (6.77)	**1.21 (1.02, 1.42)**, ***p* = 0.024**	–
Number of START PPOs			
0 START (*n* = 1937)	1.66 (6.27)	–	(Referent)
1 START (*n* = 650)	2.11 (8.07)	–	**1.26 (1.04, 1.52)**, ***p* = 0.018**
≥ 2 START (*n* = 412)	2.06 (3.93)	–	1.13 (0.91, 1.41), *p* = 0.271

	*M* (SD)^a^	Hospital admission (*n* = 2501) Adjusted IRR (95% CI)^b^	
		Model 1^c^	Model 2^d^
Any STOPP^e^ (*n* = 916)	0.49 (1.79)	**1.38 (1.08, 1.75)**, ***p* = 0.009**	–
Number of STOPP PIMS			
0 STOPP (*n* = 2091)	0.28 (1.03)	–	(Referent)
1 STOPP (*n* = 662)	0.46 (1.92)	–	1.28 (0.99, 1.66), *p* = 0.062
≥ 2 STOPP (*n* = 254)	0.59 (1.40)	–	**1.70 (1.17, 2.47)**, ***p* = 0.006**
Any START^e^ (*n* = 1066)	0.44 (1.55)	1.24 (0.99, 1.54), *p* = 0.063	–
Number of START PPOs			
0 START (*n* = 1941)	0.29 (1.16)	–	(Referent)
1 START (*n* = 653)	0.40 (1.66)	–	1.21 (0.93, 1.57), *p* = 0.158
≥ 2 START (*n* = 413)	0.52 (1.37)	–	1.28 (0.95, 1.73), *p* = 0.108

^a^Mean and standard deviation of healthcare utilisation variables at Wave 5

^b^Adjusted for age group, sex, education, employment status, insurance coverage, multimorbidity, polypharmacy, and wave 4 healthcare utilisation

^c^Model 1: PIP exposure assessed using binary variables for presence or absence of STOPP/START

^d^Model 2: PIP exposure assessed using categorical variables for presence of 0, 1 and ≥ 2 STOPP/START criteria

^e^Referent: none

^f^Associations with p <0.05 are in bold

**Table 5:** Association between STOPP/START and the outcome of functional decline using logistic regression (odds ratio (OR), 95% confidence intervals (CIs))

	*n* (%)^a^	Functional decline (*n* = 2507) Adjusted OR (95% CI)^b^	
		Model 1^c^	Model 2^d^
Any STOPP^e^ (*n* = 917)	204 (22.25)	**1.46 (1.11, 1.91)**, ***p* = 0.007**^**f**^	–
Number of STOPP PIMS			
0 STOPP (*n* = 2095)	230 (10.98)	–	(Referent)
1 STOPP (*n* = 663)	116 (17.50)	–	1.27 (0.94, 1.72), *p* = 0.116
≥ 2 STOPP (*n* = 254)	88 (34.65)	–	**2.02 (1.36, 2.99)**, ***p* < 0.001**
Any START^e^ (*n* = 1067)	212 (19.87)	1.23 (0.95, 1.60), *p* = 0.114	–
Number of START PPOs			
0 START (*n* = 1945)	222 (11.41)	–	(Referent)
1 START (*n* = 653)	120 (18.38)	–	1.28 (0.94, 1.73), *p* = 0.110
≥ 2 START (*n* = 414)	92 (22.22)	–	1.17 (0.83, 1.64), *p* = 0.364

^a^Number and percentage who experienced functional decline at Wave 5, referent: none

^b^Adjusted for age group, sex, education, employment, insurance coverage, multimorbidity, polypharmacy, and functional impairment at Wave 4

^c^Model 1: PIP exposure assessed using binary variables for presence or absence of STOPP/START

^d^Model 2: PIP exposure assessed using categorical variables for presence of 0, 1 and ≥ 2 STOPP/START criteria

^e^Referent: none

^f^Associations with p <0.05 are in bold

## Discussion

### Principal findings

Approximately one-third of study participants experienced one or more STOPP v3-defined PIMs. The majority of these had 1 STOPP PIM, with 8.5% of the total experiencing 2 STOPP PIMs. The most common examples of STOPP PIMs in this cohort were aspirin prescribed for the primary prevention of cardiovascular disease and duplicate drug class prescriptions.

More than one-third of study participants met START PPO criteria, reflecting an omission of clinically indicated medications. The most common PPOs were ACEI omitted in the context of known cardiovascular disease and vitamin D omission for patients with known osteoporosis. Of note, 7.4% of study participants met the criteria for depression using a validated scale but were not prescribed an antidepressant medication, indicating potentially unmet clinical need in this area.

Increasing age, female sex and polypharmacy were associated with an increased risk of STOPP PIMs. Hyperpolypharmacy was associated with a ninefold increased risk of STOPP PIMs compared to participants not experiencing polypharmacy. Over the follow-up period, participants with STOPP PIMs were significantly more likely to be admitted to hospital and to experience functional decline. Patients with START-defined prescribing omissions reported more GP, outpatient and ED visits over time, but there was no evidence of an association with emergency admissions or functional decline.

### Comparison with other studies

Previous international longitudinal studies examining the prevalence of earlier versions of STOPP/START criteria found generally higher prevalence rates of PIMs and PPOs than the current study. In a systematic review (including 30 non-randomised studies) the prevalence of STOPP PIMs (version 2) was 42% in community settings and 51% in hospital settings [26]. Another systematic review reported prevalence rates of STOPP PIMs (version 1) as ranging between 21 and 79% due to significant heterogeneity in study design and study populations [27]. In a previous study using the same longitudinal cohort as the current study, with linked pharmacy claims data, rates of STOPP v2 PIMs and START v2 PPOs were 57% and 41% respectively [19]. The current study reported rates of STOPP v3 PIMs at 31% and START v3 PPOs at 36% and therefore suggest an improvement in prescribing appropriateness over time. It is important to note when comparing the two studies that there was overlap in less than 50% of the STOPP criteria applied and in one-third of START criteria applied. However, there has been a substantial expansion of the numbers of STOPP/START version 3 criteria reflecting the growth in the pharmacotherapy evidence base and medication availability over time. It must also be borne in mind that in the current study only 45% of STOPP criteria and 28% of START criteria could be applied due to the limited available data.

The current study found that older patient age, female sex and increasing number of medications are factors associated with an increased risk of STOPP PIMs, in line with the results of previous studies [19, 28]. Balancing the risks and benefits of multiple medications to treat multimorbidity with increasing age-related physiological changes requires constant trade-offs. Patients can experience high levels of treatment burden adhering to complex medication regimes from attending several healthcare professionals across different settings [29]. For clinicians, balancing the risk-to-benefit tipping point for individual patients is often very challenging and requires integration of available evidence, clinical experience and patient preferences [30].

The consistent emergence of female sex as a predictor of PIM occurrence may have implications for developing targeted interventions to address PIM use [28, 31, 32]. Both sex and gender may influence likelihood for PIMs via several mechanisms including biological differences in disease manifestation, differing pharmacokinetic and pharmacodynamic responses, as well as differences in both healthcare seeking behaviour and prescriber behaviour [33]. Women are more likely to be prescribed psychotropic medication and in Ireland rates of benzodiazepine and z-drug hypnotic prescribing was highest for older (≥ 65 years) Irish women over a ten-year period [34, 35]. Disaggregation of data to examine how sex and gender intersect with PIP may reveal further insights from which targeted interventions may be developed [33, 36].

In this study START PPOs were associated with increasing age (≥ 75 years) and patients living with three or more long-term conditions were most at risk. PPOs in this cohort may reflect some of the difficulties of applying clinical guideline recommendations that focus on single diseases to this group. Single disease focussed clinical guidelines are underpinned by evidence that often excludes older people with multimorbidity. Thus, applying these guidelines for this group can drive polypharmacy, in the absence of guidance on how best to prioritise treatment recommendations [29, 37]. Given these difficulties, clinicians should carefully consider the balance of pharmacotherapy recommendations to avoid the ‘risk/treatment paradox’ (higher‐risk older patients denied safe medications likely to improve survival or health-related quality of life) whilst avoiding potentially inappropriate use of medications for which risks are likely to outweigh benefits [38].

This study also found that patients in receipt of one or more STOPP PIMs were more likely to experience functional decline over time and more likely to experience an emergency hospital admission compared to participants not exposed to STOPP PIMs. This aligns with the findings of a 2019 systematic review and meta-analysis (*n* = 4 studies) which reported that the presence of STOPP PIMs was associated with a range of adverse outcomes including increased healthcare utilisation, adverse drug events and functional decline [39].

There is limited existing research examining START PPOs and subsequent health outcomes, however [8]. In the present study, START PPOs were associated with an increase in GP, outpatient and ED visits, indicating potentially unmet clinical need in this group. There was no impact on emergency admission or functional decline.

### Implications for clinical practise, policy and research

A considerable proportion of NSAID and z-drug users at Wave 4 also reported use of these drug classes at Wave 3, suggesting continuous use. This is despite the known gastrointestinal, renal, cardiovascular and cerebrovascular risks associated with chronic NSAID use and prescribing advice to use the lowest effective dose for the shortest possible time [40]. Similarly, chronic z-drug use (≥ 2 weeks) increases the risk of falls and fractures, which can have potentially devastating consequences for older adults [41]. This finding aligns with a previous Irish study-in 2019, 27% of all patients dispensed z-drugs received these for three months or more, an increase from 22% in 2015 [42]. Deprescribing opportunities were identified in participants aged ≥ 85 years with established frailty who used statins for primary prevention. Evidence for statin efficacy in the ‘old old’ age cohort is uncertain and therefore statins should only be considered on an individual basis considering level of cardiovascular risk and life expectancy [43, 44].

Addressing PIP may also confer healthcare system benefits through a reduction in healthcare attendances at a time when global healthcare systems face increasing demand. Reducing the number of unplanned hospital admissions for older patients may in turn reduce the likelihood for further PIP, as transitions of care are a key risk factor for medication-related harm [45, 46]. One challenge with the application of the STOPP/START version 3 is the large number of criteria to be applied (*n* = 190). Automating prescribing indicators through integration with patient electronic record systems to alert prescribers to PIMs and PPOs is one way of addressing this issue [47].

Highlighting international concerns regarding medication safety the World Health Organisation launched a global patient safety challenge, Medication Without Harm, in March 2017 [46]. However, to date, multi-centre randomised controlled trials with interventions focussed on STOPP/START criteria and other prescribing indicators have not resulted in improvements in primary end-points such as ADRs, emergency admission and re-admission [48–51]. Recently, polypharmacy stewardship has been suggested as a novel way to approach this issue. This is defined as a “*coordinated intervention designed to assess, monitor, improve, and measure the pharmacotherapeutic treatment of multimorbidity………with the aim of aligning treatment regimens with the overall condition, prognosis, and preferences of the individual patient*” [52]. Core principles of polypharmacy stewardship include optimisation of prescribing (e.g. dose and frequency), setting of realistic treatment goals, ADR avoidance and provision of support and follow-up to patients with multimorbidity. Explicit prescribing indicator sets, such as STOPP/START criteria, have a role in identifying medicines that merit clinical review in the context of the individual patient’s clinical history and preferences, but broader healthcare system issues must also be addressed to optimise prescribing. This includes timely sharing of medication information and medicines reconciliation across fragmented healthcare settings and prioritising continuity of care [29, 30].

### Strengths and limitations

This study has several strengths. It is a nationally representative study of the Irish population and longitudinal in design allowing for follow-up over time. Comprehensive information was collected on a range of demographic, socioeconomic and clinical characteristics through rigorous standardised protocols, allowing for baseline differences to be accounted for. Medication data, collected by in-home computer-assisted participant interview (CAPI), demonstrates both internal and external validity [21]. The study has some limitations also. Similar to previous studies, only a subset of the STOPP/START v3 criteria could be applied to the TILDA dataset due to the absence of certain clinical data required for application [19, 27]. The outcomes of healthcare utilization and functional decline were self-reported which may have affected accuracy. However, adjustment for the range of confounders should have addressed any systematic reporting bias amongst participant subgroups [53]. The proportion of study participants with complex polypharmacy (10 or more medications) was low at 6%. Finally, as this is a retrospective cohort study, results of association and outcome analyses should not be regarded as causal as there are potential confounders that could not be controlled for, that could affect these associations.

## Conclusion

In this nationally representative longitudinal study, approximately one-third of participants were found to experience STOPP-defined PIMs and over one-third START-defined PPOs, when assessed using the recently updated STOPP/START prescribing indicator set. The presence of STOPP PIMs was associated with an increased risk of functional decline and hospital admission at follow-up. Increasing age and number of chronic health conditions were associated with START PPOs, highlighting the challenges in balancing the risk-to-benefit ratio of medications in older people with multimorbidity.

## Supplementary Information

Below is the link to the electronic supplementary material.

Supplementary file1 (DOCX 27 KB)

Supplementary file2 (DOCX 64 KB)

Supplementary file3 (DOCX 22 KB)

Supplementary file4 (DOCX 20 KB)

## Data Availability

Access to data from the TILDA study is granted upon approval of the request by the TILDA Data Access Committee. More information can be obtained at https://www.tilda.tcd.ie/. The publicly accessible dataset files are available via the Irish Social Science Data Archive (ISSDA) based in University College Dublin. Access to the publicly available dataset files is granted following application to the ISSDA. More information can be obtained at https://www.ucd.ie/issda/data/tilda/.
