# Supplementary material for: Prevalence of potentially inappropriate prescribing in community-dwelling older adults: an application of STOPP/START version 3 to The Irish Longitudinal Study on Ageing (TILDA)
Source: Eur Geriatr Med. 2025 Apr 28;16(4):1389–402. doi: 10.1007/s41999-025-01201-3 (PMC12378767; doi:10.1007/s41999-025-01201-3)
Supplement: Supplementary file 2 — Supplementary file2 (DOCX 64 KB) [file 41999_2025_1201_MOESM2_ESM.docx]

**ADDITIONAL FILE 2**

**Table S2. Specification of STOPP applied to TILDA Wave 4 data**

| **Indicator** | **Reason for exclusion** | **Data operationalisation** | **Drug/disease prevalence denominator** |
| --- | --- | --- | --- |
| **Section A: Indication of medication** |  |  |  |
| 1.  Any drug prescribed without a clinical indication. | Insufficient data | - | - |
| 2.  Any drug prescribed beyond the recommended duration, where treatment duration is well defined. | - | Use at two consecutive waves defined as continued exposure e.g. use at Wave 3 and Wave 4. Examined for z-drugs (N05CF) and NSAIDs (M01A) | Total number of participants reporting ATC codes:  z-drug (N05CF)  NSAIDs (M01A) |
| 3.  Any duplicate drug class prescription for daily regular use (as distinct from PRN use) e.g., two concurrent NSAIDs, SSRIs, loop diuretics, ACE inhibitors, anticoagulants, antipsychotics, opioid analgesics (optimisation of monotherapy within a single drug class should be observed prior to considering a new agent). | - | Prescription of two ATC codes within the same classification for the following: NSAIDS (M01A); SSRIs (N06AB); loop diuretics (C03C); ACEIs (C09A, C09B); anticoagulants (B01AA, B01AB, B01AE, B01AF, B01AX); antipsychotics (N05A); opioid analgesics (N02A); simulant laxatives (A06AB06; A03AB08; A06AA02; A06AB02); thiazides (C03A); beta blockers (C07); ARBs (C09C, C09D); CCBS (C08); statins (C10); benzodazepines (N05BA, N05CD); tricyclic antidepressants (N06AA) | Total number of participants reporting one ATC code within the relevant classification |
| **Section B: Cardiovascular system** |  |  |  |
| 1.  Digoxin for heart failure with normal systolic ventricular function (no clear evidence of benefit) | Insufficient data | - | - |
| 2.  Verapamil or diltiazem with NYHA Class III or IV heart failure (may worsen heart failure with reduced ejection fraction i.e., HFREF). | Insufficient data | - | - |
| 3.  Beta-blocker in combination with verapamil or diltiazem (risk of heart block). | - | Use of ATC C07 in combination with either C08DA01, C08DB01, CA08DA51 or C09BB10 | All participants who use either verapamil, diltiazem or combination medications (CA08DA51 or C09BB10) |
| 4.  Ventricular rate-limiting drugs i.e., beta blocker, verapamil, diltiazem, digoxin with bradycardia (< 50/min), type II heart block or complete heart block (risk of complete heart block, asystole). | Insufficient data | - | - |
| 5.  Beta-blocker as monotherapy for uncomplicated hypertension i.e., not associated with angina pectoris, aortic aneurysm or other condition where beta-blocker therapy is indicated (no firm evidence of efficacy). | - | Use of C07 in those with hypertension and no history of angina, heart attack, heart failure, heart rhythm disorder or atrial fibrillation at any wave and who are not prescribed ATC groups C02, C03, C08 or C09 | All participants with hypertension and no history of angina, heart attack, heart failure, heart rhythm disorder or atrial fibrillation at any wave |
| 6.  Amiodarone as first-line antiarrhythmic therapy in supraventricular tachyarrhythmias (higher risk of major side-effects than beta-blockers, digoxin, verapamil or diltiazem). | Insufficient data | - | - |
| 7.  Loop diuretic as first-line treatment for hypertension unless there is concurrent heart failure requiring diuretic therapy (safer, more effective alternatives available). | - | Use of ATC group C03CA in those with hypertension and no record of heart failure at any prior wave, as well as no record of a non-loop diuretic antihypertensive at any prior wave. | All participants with hypertension at Wave 4 and no record if heart failure at any prior wave. |
| 8.  Loop diuretic for dependent ankle oedema without clinical, biochemical or radiological evidence of heart failure, liver failure, nephrotic syndrome or renal failure (leg elevation and /or compression hosiery usually more appropriate). | Insufficient data | - | - |
| 9.  Thiazide diuretic with current significant hypokalaemia (i.e., serum K+ < 3.0 mmol/l), hyponatraemia (i.e., serum Na+ < 130 mmol/l) hypercalcaemia (i.e., corrected serum calcium > 2.65 mmol/l) or with a history of gout (hypokalaemia, hyponatraemia, hypercalcaemia and gout can be precipitated by thiazide diuretic) | - | Examined as thiazide diuretic in those with a history of gout only. Use of ATC group M04A in those who had a record of gout medication (ATC M04A) at any prior wave | All participants with a record of gout medication use at any prior wave |
| 10. Loop diuretic for treatment of hypertension with concurrent urinary incontinence (may exacerbate incontinence). | - | Use of ATC group C03CA in those with hypertension and a concurrent record of incontinence and no record of heart failure at Wave 4. | All participants with a record of hypertension and concurrent incontinence. |
| 11. Centrally-acting antihypertensives e.g., methyldopa, clonidine, moxonidine, rilmenidine, guanfacine | - | Use of ATC group C02A | All participants with a record of hypertension. |
| 12. Angiotensin-Converting Enzyme inhibitors (ACEIs) or Angiotensin Receptor Blockers (ARBs) in patients with hyperkalaemia i.e., serum K > 5.5 mmol/l. | Insufficient data | - | - |
| 13. Aldosterone antagonists (e.g., spironolactone, eplerenone) with concurrent potassiumconserving drugs (e.g., ACEI’s, ARB’s, amiloride, triamterene) without monitoring of serum potassium (risk of dangerous hyperkalaemia i.e., > 6.0 mmol/l – serum K should be monitored regularly, i.e., at least every 6 months). | Insufficient data | - | - |
| 14. Phosphodiesterase type-5 inhibitors (e.g., sildenafil, tadalafil, vardenafil) in severe heart failure characterised by hypotension i.e., systolic BP < 90 mmHg, or concurrent nitrate therapy for angina (risk of cardiovascular collapse). | - | Use of ATC codes G04BE10; G04BE11; G04BE03; G04BE08; or G04BE09) and concomitant use of nitrate (ATC group C01DA) | All participants with a record of nitrate use (ATC C01DA) |
| 15. Drugs that predictably prolong the QTc interval (QTc = QT/RR) in patients with known with demonstrable QTc prolongation (to >450 msec in males and >470 msec in females), including quinolones, macrolides, ondansetron, citalopram (doses > 20 mg/day), escitalopram (doses > 10 mg/day), tricyclic antidepressants, lithium, haloperidol, digoxin, class 1A antiarrhythmics, class III antiarrhythmics, tizanidine, phenothiazines, astemizole, mirabegron (risk of life threatening ventricular arrhythmias). | Insufficient data | - | - |
| 16. Statins for primary cardiovascular prevention in persons aged ≥ 85 and established frailty with expected life expectancy likely less than 3 years (lack of evidence of efficacy). | - | Use of ATC group C10AA with no record of heart attack, angina, anstroke, transient ischemic attack, heart failure, or atrial fibrillation at any prior wave, where the participant is 85 years or older and defined as frail using the FRAIL scale (score ≥3) | All participants with no prior heart attack, angina, stroke, transient ischemic attack, heart failure or atrial fibrillation at any prior wave, who are 85 years and older and defined as frail using the FRAIL scale (score ≥3) |
| 17. Long-term systemic i.e., non-topical NSAIDs with known history of coronary, cerebral or peripheral vascular disease (increased risk of thrombosis). | - | Use at Wave 3 and Wave 4 assumed to be continuous use. Use of ATC group M01A at waves 3 and 4, and a record of either a heart attack, angina, heart failure, atrial fibrillation, stroke or transient ischaemic attack at any prior wave | All participants with a record of either a heart attack, angina, heart failure, atrial fibrillation, stroke or transient ischaemic attack at any prior wave |
| 18. Long-term antipsychotics with known history of coronary, cerebral or peripheral vascular disease (increased risk of thrombosis). | - | Use at Wave 3 and Wave 4 assumed to be continuous use. Use of ATC group N05A at waves 3 and 4, and a record of either a heart attack, angina, heart failure, atrial fibrillation, stroke or transient ischaemic attack at any prior wave | All participants with a record of either a heart attack, angina, heart failure, atrial fibrillation, stroke or transient ischaemic attack at any prior wave |
| 19. NSAIDs or systemic corticosteroids with heart failure requiring loop diuretic therapy (risk of exacerbation of heart failure). | - | Use of ATC group M01A or systemic corticosteroid (ATC group H02AA or H02AB) in those with a record of heart failure and concomitant use of a loop diuretic (ATC group C03CA) at Wave 4. | All participants with a record of heart failure and a loop diuretic at Wave 4. |
| 20. Antihypertensive drugs in severe symptomatic aortic stenosis (risk of severe hypotension, syncope). | Insufficient data | - | - |
| 21. Digoxin as first line treatment for long-term (> 3 months) ventricular rate control in atrial fibrillation (increased mortality from long-term digoxin use; cardio-selective beta-blockers are generally preferable). | Insufficient data | - | - |
| **Section C: Coagulation System** |  |  |  |
| 1. Long-term aspirin at doses greater than 100mg per day (increased risk of bleeding, no evidence for increased efficacy). | Insufficient data | - | - |
| 2. Antiplatelet agents, vitamin K antagonists, direct thrombin inhibitors or factor Xa inhibitors with concurrent significant risk of major bleeding, i.e. uncontrolled severe hypertension, bleeding diathesis, recent non-trivial spontaneous bleeding (high risk of bleeding). | Insufficient data | - | - |
| 3. Aspirin plus clopidogrel as long-term secondary stroke prevention i.e., > 4 weeks, unless the patient has a coronary stent(s) inserted in the previous 12 months or concurrent acute coronary syndrome or has a high grade symptomatic carotid arterial stenosis (no evidence of added long-term benefit over clopidogrel monotherapy). | Insufficient data | - | - |
| 4. Antiplatelet agents in combination with vitamin K antagonist, direct thrombin inhibitor or factor Xa inhibitors in patients with chronic atrial fibrillation, unless there is concurrent coronary artery stent(s) inserted or angiographically proven high grade (> 50%) coronary artery stenosis (no added benefit from antiplatelet agents). | Insufficient data | - | - |
| 5. Antiplatelet agents with vitamin K antagonist, direct thrombin inhibitor or factor Xa inhibitors in patients with stable coronary, cerebrovascular or peripheral arterial disease (no evidence of added benefit from dual therapy). | Insufficient data | - | - |
| 6. Ticlopidine in any circumstances (clopidogrel and prasugrel have similar efficacy, stronger evidence and fewer side-effects). | - | Use of ticlodipine (ATC B01AC05) | - |
| 7. Antiplatelet agents as alternatives to vitamin K antagonists, direct thrombin inhibitors or factor Xa inhibitors for stroke prevention in patients with chronic atrial fibrillation (no evidence of efficacy). | - | Use of ATC group B01AC in those with a record of atrial fibrillation at Wave 4 and no record of stroke or transient ischaemic attack at any prior wave, and with no use of ATC groups (B01AA; B01AE; or B01AF) | All participants with a record of atrial fibrillation at Wave 4 and no record of stroke or transient ischaemic attack at any prior wave |
| 8. Vitamin K antagonist, direct thrombin inhibitor or factor Xa inhibitors for first deep venous thrombosis without continuing provoking risk factors (e.g., thrombophilia) for longer than 6 months, (no proven added benefit). | Insufficient data | - | - |
| 9.      Vitamin K antagonist, direct thrombin inhibitor or factor Xa inhibitors for first pulmonary embolus without continuing provoking risk factors (e.g., thrombophilia) for longer than 12 months (no proven added benefit). | Insufficient data | - | - |
| 10.  Nonsteroidal anti-inflammatory drugs (NSAIDs) and vitamin K antagonist, direct thrombin inhibitor or factor Xa inhibitors in combination (risk of major gastrointestinal bleeding). | - | Use of ATC group M01A and concomitant use of ATC group B01AA; B01AE or B01AF at Wave 4 | All participants with a record of ATC group B01AA; B01AE or B01AF at Wave 4 |
| 11.  Vitamin K antagonist as first-line anticoagulant for atrial fibrillation, unless there is concurrent metallic heart valve in-situ, moderate-to-severe mitral stenosis, or eGFR < 15 mls/min./1.73m^2^ (direct thrombin inhibitors or factor Xa inhibitors are equally efficacious and safer than vitamin K antagonists). | Insufficient data | - | - |
| 12.  Selective serotonin reuptake inhibitors (SSRIs) in combination with Vitamin K antagonist, direct thrombin inhibitor or factor Xa inhibitor with a previous history of major haemorrhage (increased risk of bleeding due to antiplatelet effects of SSRIs). | Insufficient data | - | - |
| 13.  Direct thrombin inhibitor (e.g., dabigatran) and diltiazem or verapamil (increased risk of bleeding). | - | Use of ATC group B01AE in combination with either C08DA01, C08DB01, CA08DA51 or C09BB10 | All participants with a record of either C08DA01, C08DB01, CA08DA51 or C09BB10 at Wave 4 |
| 14.  Apixaban, dabigatran, edoxaban, rivaroxaban and P-glycoprotein (P-gp) drug efflux pump inhibitors e.g., amiodarone, azithromycin, carvedilol, cyclosporin, dronedarone, itraconazole, ketoconazole (systemic), macrolides, quinine, ranolazine, tamoxifen, ticagrelor, verapamil (increased risk of bleeding). | - | Use of ATC code B01AF02; B01AE07; B01AF03; B01AF01; or B01AF51 in combination with a P-glycoprotein pump inhibitor (C01BD01; J01AF10; C07AG02; L04AD01; C01BD07; J02AC02; J02AB02; J01FA; P01BC01; C01EB18; L02BA01; B01AC24; C08DA01) | All participants with a record of a P-glycoprotein inhibitor at Wave 4 |
| 15.  Systemic oestrogens or androgens with pervious history of venous thromboembolism (increased risk of recurrent venous thromboembolism). | Insufficient data | - | - |
| 16.  Aspirin for primary prevention in cardiovascular disease (no evidence of benefit). | - | Use of ATC code B01AC06 in those with no record of heart attack, angina, heart failure, stroke, transient ischemic attack, or atrial fibrillation at any prior wave | All participants with no record of heart attack, angina, heart failure, stroke, transient ischemic attack, or atrial fibrillation at any prior wave |
| **Section D: Central Nervous System** |  |  |  |
| 1. TriCyclic Antidepressants (TCAs) in patients with dementia, narrow angle glaucoma, cardiac conduction abnormalities, prostatism, chronic constipation, recent falls, prior history of urinary retention or orthostatic hypotension (risk of worsening these conditions). | - | Use of ATC group N06AA, in those with Alzheimer’s disease, dementia or other serious cognitive impairment, OR glaucoma, OR experienced a fall in the 12 months preceding the Wave 4 interview or since the Wave 3 interview | All participants with cognitive impairment, glaucoma or a fall in the 12 months preceding the Wave 4 interview or since the Wave 3 interview |
| 2. Initiation of TriCyclic Antidepressants (TCAs) as first-line treatment for major depression (higher risk of adverse drug reactions with TCAs than with SSRIs or SNRIs). | Insufficient data | - | - |
| 3. Serotonin/noradrenaline reuptake inhibitors (SNRI’s e.g., venlafaxine, duloxetine) and severe hypertension i.e., systolic blood pressure > 180 mmHg +/- diastolic blood pressure > 105 mmHg (likely to make hypertension worse). | Insufficient data | - | - |
| 4. Antipsychotics with moderate-marked antimuscarinic/anticholinergic effects (acepromazine, chlorpromazine, clozapine, flupenthixol, fluphenzine, levomepromazine, olanzapine, pipothiazine, promazine, thioridazine) with a history of lower urinary tract symptoms associated with benign prostatic hyperplasia or previous urinary retention (high risk of urinary retention). | Insufficient data | - | - |
| 5. Antipsychotics prescribed for behavioural and psychological symptoms of dementia (BPSD) an unchanged dose for > 3 months without medication review (increased risk of extrapyramidal side-effects and chronic worsening of cognition, increased risk of major cardiovascular morbidity and mortality). | Insufficient data | - | - |
| 6. Selective serotonin re-uptake inhibitors (SSRI’s) with current or recent significant hyponatraemia i.e., serum Na+ < 130 mmol/l (risk of exacerbating or precipitating hyponatraemia). | Insufficient data | - | - |
| 7. Selective serotonin re-uptake inhibitors (SSRI’s) with current or recent significant bleeding (risk of exacerbation or recurrence of bleeding due to antiplatelet effects of SSRI’s). | Insufficient data | - | - |
| 8. Benzodiazepines for ≥ 4 weeks (no indication for longer treatment; risk of prolonged sedation, confusion, impaired balance, falls, road traffic accidents; all benzodiazepines should be withdrawn gradually if taken for more than 4 weeks as there is a risk of causing a benzodiazepine withdrawal syndrome if stopped abruptly). | - | Use of ATC groups N05BA or N05CD at Wave 4 and Wave 3 assumed continuous use | All participants who use N05BA or N05CD at Wave 4 |
| 9. Benzodiazepines for agitated behaviour or psychotic symptoms of dementia (no evidence of efficacy). | - | Use of ATC groups N05BA or N05CD in those with Alzheimer’s disease, dementia or other serious cognitive impairment and with no record of sleep disturbance or anxiety at Wave 4 | All participants with a record of Alzheimer’s disease, dementia or other serious cognitive impairment and with no record of sleep disturbance or anxiety at Wave 4 |
| 10. Benzodiazepines for insomnia for ≥ 2 weeks (high risk of dependency, increased risk of falls, fractures and road traffic accidents). | - | Use of ATC groups N05BA or N05CD at both Wave 4 and Wave 3 assumed continuous use. Use of benzodiazepines at Wave 3 and Wave 4, in those with a record of sleep disturbance at Wave 3 and Wave 4 | All participants with a record of sleep disturbance at Wave 3 and Wave 4 |
| 11. Z-drugs (zolpidem, zopiclone, zaleplon) for insomnia for ≥ 2 weeks (increased risk of falls, fractures). | - | Use of ATC group N05CF at Wave 4 and Wave 3 assumed continuous use. Use of NO5CF at Wave 3 and Wave 4, in those with a record of sleep disturbance at Wave 3 and Wave 4 | All participants with a record of sleep disturbance at Wave 3 and Wave 4 |
| 12. Antipsychotics (i.e., other than clozapine or quetiapine) in those with parkinsonism or Dementia with Lewy Bodies (risk of severe extra-pyramidal symptoms). | - | Use of ATC group N05A, but not N05AH02 or N05AH04, in those with a record of Parkinson’s disease at Wave 4 | All participants with a record of Parkinson’s disease at Wave 4 |
| 13. Anticholinergic/antimuscarinic drugs (biperiden, orphenadrine, procyclidine, trihexyphenidyl) to treat extra-pyramidal side-effects of antipsychotic medications (risk of anticholinergic toxicity). | - | Use of ATC codes N04AA02, N04AB02, N04AA04, or N04AA01 and antipsychotics (ATC group N05A) at Wave 4, with use of antipsychotic only at wave 3, and with no record of Parkinson’s disease at Wave 4 | All participants with use of an antipsychotic (N05A) at Wave 4 |
| 14. Drugs with potent anticholinergics/antimuscarinic effects** in patients with delirium or dementia (risk of exacerbation of cognitive impairment). | - | Use of amitriptyline (N06AA09) OR doxepin (N06AA12) OR imipramine (N06AA02) OR nortriptyline (N06AA10) OR chlorpromazine (N05AA01) OR clozapine (N05AH02) OR thioridazine (N05AC02) OR first generation antihistamines(R06AA or R06AB or R06AC or RO6AD) OR urinary anticholinergics (G04BD02, G04BD04, G04BD06, G04BD07, G04BD08, G04BD09, G04BD10, G04BD11) OR hyoscine (A03AB05) OR procyclidine (N04AA04) OR benzatropine (N04AC01) OR tizanidine (M03BX02), in those with Alzheimer’s disease, dementia or other serious cognitive impairment at Wave 4 | All participants with Alzheimer’s disease, dementia or other serious cognitive impairment at Wave 4 |
| 15.  Antipsychotics in patients with behavioural and psychological symptoms of dementia (BPSD) for longer than 12 weeks unless BPSD symptoms are severe and other nonpharmacological treatments have failed (increased risk of stroke, myocardial infarction). | Insufficient data | - | - |
| 16.  Antipsychotics as hypnotics, unless sleep disorder is due to psychosis or BPSD effects of dementia (not recommended in summary of product characteristics; increased risk of confusion, hypotension, extra-pyramidal side effects, falls). | - | Use of ATC group N05A, in those with a record of sleep disturbance at Wave 4, and no record of psychosis, schizophrenia, Alzheimer’s disease, dementia or other serious cognitive impairment | All participants with a record of sleep disturbance at Wave 4, and no record of psychosis, schizophrenia, Alzheimer’s disease, dementia or other serious cognitive impairment |
| 17.  Acetylcholinesterase inhibitors with a known history of persistent bradycardia (< 60 beats/min.), heart block or recurrent unexplained syncope (risk of cardiac conduction failure, syncope and injury). | Insufficient data | - | - |
| 18.  Acetylcholinesterase inhibitors with concurrent treatment with drugs that induce persistent bradycardia (< 60 beats/min.) such as beta-blockers, digoxin, diltiazem, verapamil (risk of cardiac conduction failure, syncope and injury). | - | Use of ATC group N06D in combination with beta blockers (C07), digoxin (C01AA05), diltiazem or verapamil (C08DA01; C08DB01; C08DA51; C09BB10) | All participants with use of a beta blocker, digoxin or verapamil at Wave 4 |
| 19.  Memantine with known current or previous seizure disorder (increased risk of seizures). | - | Use of ATC codes N06DX01, N06DA52, or N06DA53 in those with a record of epilepsy at Wave 4 | All participants who have a record of epilepsy at Wave 4 |
| 20.  Nootropics in dementia including Gingko Biloba, piracetam, pramiracetam, phenylpiracetam, aniracetam, phosphatidylserine, modafinil, L-theanine, omega-3 fatty acids, panax ginseng, rhodiola, creatine (no evidence of efficacy). | - | Restricted to only those nootropics with an ATC code. Use of ATC codes N06BX03, N06BX11, N06BA07 or C10AX06 in those with a record of Alzheimer’s disease, dementia or other serious cognitive impairment at Wave 4 | All participants with a record of Alzheimer’s disease, dementia or other serious cognitive impairment at Wave 4 |
| 21.  Phenothiazines as first-line treatment for psychosis or non-cognitive symptoms of dementia, since safer and more efficacious alternatives exist (phenothiazines are sedative, have significant anti-muscarinic toxicity in older people, except for prochlorperazine for nausea/vomiting/vertigo, chlorpromazine for relief of persistent hiccoughs and levomepromazine as an anti-emetic in palliative care). | Insufficient data | - | - |
| 22.  Levodopa or dopamine agonists for benign essential tremor (no evidence of efficacy) | Insufficient data | - | - |
| 23.  Levodopa or dopamine agonists for treatment of extrapyramidal side-effects of antipsychotics or other forms of drug-induced Parkinsonism (inappropriate prescribing cascade to be avoided). | - | Use of levodopa (N04BA01) OR dopamine agonist (N04BC) AND antipsychotic (N05A) at Wave 4 with use of antipsychotic only at Wave 3, and with no record of Parkinson’s disease at Wave 4 | All participants with use of an antipsychotic (N05A) at Wave 4 |
| 24.  First-generation antihistamines as first-line treatment for allergy or pruritus (safer antihistamines with fewer side-effects now widely available). | Insufficient data | - | - |
| 25.  First-generation antihistamines for insomnia (high risk of side-effects, Z-drugs safer and more appropriate for short-term use). | Insufficient data | - | - |
| **Section E: Renal System** |  |  |  |
| 1. Digoxin at a long-term (i.e. more than 90 days) maintenance dose ≥ 125µg/day if eGFR < 30 ml/min/1.73m^2^ (risk of digoxin toxicity if plasma levels not measured). | Insufficient data | - | - |
| 2. Direct thrombin inhibitors (e.g., dabigatran) if eGFR < 30 ml/min/1.73m^2^ (risk of bleeding) | Insufficient data | - | - |
| 3.  Factor Xa inhibitors (e.g., rivaroxaban, apixaban, edoxaban) if eGFR < 15 ml/min/1.73m^2^ (risk of bleeding) | Insufficient data | - | - |
| 4. NSAID’s if eGFR < 50 ml/min/1.73m^2^ (risk of deterioration in renal function). | Insufficient data | - | - |
| 5. Colchicine if eGFR < 10 ml/min/1.73m^2^ (risk of colchicine toxicity) | Insufficient data | - | - |
| 6. Metformin if eGFR < 30 ml/min/1.73m^2^ (risk of lactic acidosis). | Insufficient data | - | - |
| 7. Mineralocorticoid receptor antagonists (e.g. spironolactone, eplerenone) if eGFR < 30 ml/min/1.73m^2^ (risk of dangerous hyperkalaemia). | Insufficient data | - | - |
| 8.  Nitrofurantoin if eGFR < 45 ml/min/1.73m^2^ (increased risk of nitrofurantoin toxicity). | Insufficient data | - | - |
| 9. Bisphosphonates if eGFR<30 ml/min/1.73m^2^ (increased risk of acute renal failure). | Insufficient data | - | - |
| 10.  Methotrexate if eGFR <30 ml/min/1.73m^2^ (increased risk of methotrexate toxicity). | Insufficient data | - | - |
| **Section F: Gastrointestinal System** |  |  |  |
| 1.  Prochlorperazine or metoclopramide with Parkinsonism (risk of exacerbating Parkinsonian symptoms). |  | Use of prochlorperazine (N05AB04) or metoclopramide (A03FA01) in those with a record of Parkinson's at Wave 4 | All participants with a record of Parkinson's at Wave 4 |
| 2.  Proton pump inhibitor (PPI) for uncomplicated peptic ulcer disease at full therapeutic dosage for > 8 weeks (dose reduction or earlier discontinuation or H2 antagonist maintenance therapy usually indicated). | Insufficient data | - | - |
| 3.  Drugs likely to cause constipation (e.g. systemic antimuscarinics, oral iron, opioids, verapamil, aluminium antacids) with chronic constipation where non-constipating alternatives are available (risk of exacerbation of constipation). | Insufficient data | - | - |
| 4.  Oral elemental iron doses greater than 200 mg daily (e.g. ferrous fumarate> 600 mg/day, ferrous sulphate > 600 mg/day, ferrous gluconate> 1800 mg/day; no evidence of enhanced iron absorption above these doses). | Insufficient data | - | - |
| 5.  Corticosteroids with a history of peptic ulcer disease or erosive oesophagitis (risk of relapse unless proton pump inhibitor is co-prescribed). | - | Use of systemic corticosteroids (ATC group H02AA or H02AB) in those with a record of ulcer at any prior wave, and without concomitant use of a proton pump inhibitor (ATC group A02BC) | All participants with a record of ulcer at any prior wave |
| 6.  Antiplatelet or anticoagulant drugs with a history of Gastric Antral Vascular Ectasia (GAVE, “watermelon stomach”) (risk of major gastrointestinal bleeding). | Insufficient data | - | - |
| 7.  Antipsychotics with dysphagia (increased risk of aspiration pneumonia). | Insufficient data | - | - |
| 8.  Megestrol acetate to increase appetite (increased risk of thrombosis and death with unproven efficacy) | Insufficient data | - | - |
| **Section G: Respiratory System** |  |  |  |
| 1.  Theophylline as monotherapy for COPD (safer, more effective alternative; risk of adverse effects due to narrow therapeutic index). | - | Use of theophylline (ATC code R03DA04) in those with chronic obstructive pulmonary disease (COPD) and no record of using inhalant therapy (ATC groups R03A or R03B) at Wave 4 | All participants with a record of COPD at Wave 4 |
| 2.  Systemic corticosteroids instead of inhaled corticosteroids for maintenance therapy in moderate-severe COPD (unnecessary exposure to long-term side-effects of systemic corticosteroids and effective inhaled therapies are available). | - | Use of systemic corticosteroids (ATC groups H02AA or H02AB) in those with COPD and no record of using inhalant therapy (ATC groups R03A or R03B) at Wave 4 | All participants with a record of COPD at Wave 4 |
| 3.  Long-acting muscarinic antagonists (e.g., tiotropium, aclidinium, umeclidinium, glycopyrronium) with a history of narrow angle glaucoma (may exacerbate glaucoma) or bladder outflow obstruction (may cause urinary retention). | - | Use of ATC codes R03BB04; R03BB05; R03BB07; orR03BB06) in those with a record of glaucoma at Wave 4 | All participants with a record of glaucoma at Wave 4 |
| 4.  Benzodiazepines with acute or chronic respiratory failure i.e. pO2 < 8.0 kPa ± pCO2 > 6.5 kPa (risk of exacerbation of respiratory failure). | Insufficient data | - | - |
| **Section H: Musculoskeletal System** |  |  |  |
| 1. Non-steroidal anti-inflammatory drugs (NSAIDs) other than COX-2 selective agents with history of peptic ulcer disease or gastrointestinal bleeding, unless with concurrent PPI or H2 antagonist (risk of peptic ulcer relapse). | - | Use of ATC groups M01AA, M01AB, M01AC, M01AE or M01AG but not M01AH, without concurrent use of ATC groups A02BC or A02BA, in those with a record of an ulcer at any prior wave | All participants with a record of an ulcer at any prior wave |
| 2. NSAID’s with severe hypertension i.e., systolic blood pressure consistently above 170 mmHg and/or diastolic blood pressure consistently above 100 mmHg (risk of exacerbation of hypertension). | Insufficient data | - | - |
| 3. Long-term use of NSAID (>3 months) for symptom relief of osteoarthritis pain where paracetamol has not been tried (simple analgesics preferable and usually as effective for pain relief and safer). | Insufficient data | - | - |
| 4. Long-term corticosteroids (>3 months) as monotherapy for rheumatoid arthritis (risk of systemic corticosteroid side-effects). | - | Use of systemic corticosteroid (H02AA or H02AB) at Wave 3 and Wave 4 assumed continuous use. Use of ATC groups H02AA or H02AB, in those with a record of rheumatoid arthritis at Wave 4, and no record of concurrent use of methotrexate (ATC L01BA01) or TNF alpha inhibitors (ATC group L04AB) or immunosuppressants (L04AA24, L01FA01, L04AC03, P01BA02), and no record of asthma or COPD at Wave 4 | All participants with a record of rheumatoid arthritis and with no record of asthma or COPD at Wave 4 |
| 5. Corticosteroids (other than periodic intra-articular injections for mono-articular pain) for osteoarthritis (risk of systemic corticosteroid side-effects). | - | Use of systemic corticosteroids (H02AA or H02AB), in those with a record of osteoarthritis at Wave 4, and with no record of asthma or COPD at Wave 4 | All participants with a record of osteoarthritis and with no record of asthma or COPD at Wave 4 |
| 6. Long-term NSAID or colchicine (>3 months) for chronic treatment of gout where there is no contraindication to a xanthine-oxidase inhibitor (e.g., allopurinol, febuxostat) (xanthineoxidase inhibitors are first choice prophylactic drugs in gout). | Insufficient data | - | - |
| 7. NSAID with concurrent corticosteroids for treatment of arthritis/rheumatism of any kind (increased risk of peptic ulcer disease). | - | Use of systemic corticosteroids (H02AA or H02AB) and NSAIDS (ATC group M01A) in those with a record of any kind of arthritis at Wave 4, and with no record of asthma or COPD at Wave 4 | All participants with a record of any kind of arthritis at Wave 4, and with no record of asthma or COPD at Wave 4 |
| 8. Oral bisphosphonates in patients with a current or recent history of upper gastrointestinal disease i.e. dysphagia, oesophagitis, gastritis, duodenitis, or peptic ulcer disease, or upper gastrointestinal bleeding (risk of relapse/exacerbation of oesophagitis, oesophageal ulcer, oesophageal stricture). | - | Use of ATC group M05BA in those with a record of an ulcer at any prior wave | All participants with a record of an ulcer at any prior wave |
| 9. Long-term opioids for osteoarthritis (lack of evidence of efficacy, increased risk of serious side-effects). | - | Use of ATC group N02 at Wave 3 and Wave 4 assumed continuous use. Use of N02 in those with a record of osteoarthritis at Wave 3 and Wave 4, and with no record of cancer at either Wave | All participants with a record of osteoarthritis and no record of cancer at both Wave 3 and Wave 4 |
| **Section I: Urogenital System** |  |  |  |
| 1. Systemic antimuscarinic drugs (e.g., oxybutynin, tolterodine, trospium) with dementia or chronic cognitive impairment (risk of increased confusion, agitation). | - |  |  |
| 2. Systemic antimuscarinic drugs (e.g., oxybutynin, tolterodine, trospium) with narrow-angle glaucoma (risk of acute exacerbation of glaucoma). | - |  |  |
| 3. Systemic antimuscarinic (e.g., oxybutynin, tolterodine, trospium) drugs for lower urinary tract symptoms with benign prostatic hyperplasia (BPH) and high post-void residual volume i.e. > 200 ml (uncertain efficacy and increased risk of urinary retention in older men). | Insufficient data | - | - |
| 4. Systemic antimuscarinic drugs (e.g., oxybutynin, tolterodine, trospium) with constipation (risk of exacerbation of constipation). | Insufficient data | - | - |
| 5. Alpha-1 receptor antagonists other than silodosin (e.g., alfuzosin, doxazosin, indoramin, tamsulosin, terazosin) with symptomatic orthostatic hypotension or history of syncope (risk of precipitating recurrent syncope). | Insufficient data | - | - |
| 6. Mirabegron in labile or severe hypertension (risk of exacerbation of hypertension). | Insufficient data | - | - |
| 7. Duloxetine with urinary urgency or urge incontinence (duloxetine is indicated in stress incontinence but not in urinary urgency or urge incontinence). | Insufficient data | - | - |
| 8. Antibiotic use in asymptomatic bacteriuria (no indication for treatment). | Insufficient data | - | - |
| **Section J. Endocrine System** |  |  |  |
| 1.  Sulphonylureas with a long half-life (e.g., glibenclamide, chlorpropamide, glimepiride) with type 2 diabetes mellitus (risk of prolonged hypoglycaemia). | - | Use of ATC codes A10BB01, A10BB02, A10BB12, or A01BB03 in those with a record of Type2 diabetes at Wave 4 | All participants with a record of Type 2 diabetes at Wave 4 |
| 2.  Thiazolidenediones (e.g., rosiglitazone, pioglitazone) with heart failure (risk of exacerbation of heart failure). | - | Use of ATC group A10BG in those with a record of heart failure at Wave 4 | All participants with a record of heart failure at Wave 4 |
| 3.  Non-selective beta-blockers in diabetes mellitus with frequent hypoglycaemic episodes (risk of suppressing hypoglycaemic symptoms). | Insufficient data | - | - |
| 4.  Sodium glucose co-transporter (SGLT2) inhibitors (e.g., canagliflozin, dapagliflozin, empagliflozin, ertugliflozin) with symptomatic hypotension (risk of exacerbation of hypotension). | Insufficient data | - | - |
| 5.  Systemic oestrogens with a history of breast cancer (increased risk of recurrence). | - | Use of ATC group G03C in those with a record of breast cancer recorded at any prior wave | All participants with a record of breast cancer at any prior wave |
| 6.  Systemic oestrogens with a history of venous thromboembolism (increased risk of recurrence). | Insufficient data | - | - |
| 7.  Menopausal hormone therapy (oestrogen plus progestin) with a history of stenotic coronary, cerebral or peripheral arterial disease (increased risk of acute arterial thrombosis). | Insufficient data | - | - |
| 8.  Systemic oestrogens without progestogens in patients with intact uterus (risk of endometrial cancer). | Insufficient data | - | - |
| 9.  Levothyroxine in subclinical hypothyroidism i.e., normal free T4, elevated TSH but < 10 mU/L (no evidence of benefit, risk of iatrogenic thyrotoxicosis). | Insufficient data | - | - |
| 10. Vasopressin analogues (e.g., desmopressin, vasopressin) for urinary incontinence or urinary frequency (risk of symptomatic hyponatraemia). | - | Use of ATC group H01BA in those with a record of incontinence at Wave 4 | All participants with a record of incontinence at Wave 4 |
| **Section K: Drug classes that predictably increase falls risk in susceptible older people.** |  |  |  |
| 1. Benzodiazepines in patients with recurrent falls (may cause reduced sensorium, impair balance). | - | Use of ATC groups N05BA or N05CD in those who reported two or more falls in the 12 months before Wave 4 interview or since their Wave 3 interview | All participants who recorded two or more falls in the 12 months before their Wave 4 interview or since their Wave 3 interview |
| 2. Antipsychotic drugs in patients with recurrent falls (may cause Parkinsonism). | - | Use of ATC group N05AA in those who reported two or more falls in the 12 months before Wave 4 interview or since their Wave 3 interview | All participants who recorded two or more falls in the 12 months before their Wave 4 interview or since their Wave 3 interview |
| 3. Vasodilator drugs in patients with recurrent falls with persistent postural hypotension i.e., systolic BP drop ≥ 20 mmHg and/or diastolic BP drop ≥ 10 mmHg (risk of syncope, falls). | Insufficient data | - | - |
| 4. Hypnotic Z-drugs i.e., zopiclone, zolpidem, zaleplon in patients with recurrent falls (may cause protracted daytime sedation, ataxia). | - | Use of ATC group N05CF in those who reported two or more falls in the 12 months before Wave 4 interview or since their Wave 3 interview | All participants who recorded two or more falls in the 12 months before their Wave 4 interview or since their Wave 3 interview |
| 5. Anti-epileptic drugs in patients with recurrent falls (may impair sensorium, may adversely affect cerebellar function). | - | Use of ATC group N03 in those who reported two or more falls in the 12 months before Wave 4 interview or since their Wave 3 interview | All participants who recorded two or more falls in the 12 months before their Wave 4 interview or since their Wave 3 interview |
| 6. First generation antihistamines in patients with recurrent falls (may impair sensorium). | - | Use of ATC groups R06AA, R06AB, R06AC, or RO6AD in those who reported two or more falls in the 12 months before Wave 4 interview or since their Wave 3 interview | All participants who recorded two or more falls in the 12 months before their Wave 4 interview or since their Wave 3 interview |
| 7. Opioids in patients with recurrent falls (may impair sensorium). | - | Use of ATC group N02A in those who reported two or more falls in the 12 months before Wave 4 interview or since their Wave 3 interview | All participants who recorded two or more falls in the 12 months before their Wave 4 interview or since their Wave 3 interview |
| 8. Antidepressants in patients with recurrent falls (may impair sensorium). | - | Use of ATC group N06A in those who reported two or more falls in the 12 months before Wave 4 interview or since their Wave 3 interview | All participants who recorded two or more falls in the 12 months before their Wave 4 interview or since their Wave 3 interview |
| 9.  Alpha blockers as antihypertensives in patients with recurrent falls (may cause orthostatic hypotension). | Insufficient data | - | - |
| 10. Alpha blockers for prostatic bladder outflow symptoms, other than silodosin in patients with recurrent falls (may cause orthostatic hypotension). | Insufficient data | - | - |
| 11. Centrally acting antihypertensives (may impair sensorium and may cause orthostatic hypotension). | - | Use of ATC codes C02AB01, C02AB02, C02AC01, or C02AC05 | All participants with a record of hypertension at Wave 4 |
| 12. Antimuscarinics for treatment of overactive bladder or urge incontinence (may impair sensorium). | - | Use of urinary anticholinergics (G04BD02, G04BD04, G04BD06, G04BD07, G04BD08, G04BD09, G04BD10, G04BD11) | All participants with a record of incontinence at Wave 4 |
| **Section L: Analgesic Drugs** |  |  |  |
| 1. Use of oral or transdermal strong opioids (morphine, oxycodone, fentanyl, buprenorphine, diamorphine, methadone, tramadol, pethidine, pentazocine) as first line therapy for mild pain (WHO analgesic ladder not observed; paracetamol or NSAID not prescribed as first-line therapy). | Insufficient data | - | - |
| 2. Use of daily regular (as distinct from PRN) opioids without concomitant laxative (risk of severe constipation). | Insufficient data | - | - |
| 3.  Long-acting opioids without short-acting opioids for break-through moderate or severe pain (risk of persistence of severe pain). | Insufficient data | - | - |
| 4. Topical lidocaine (lignocaine) patch for treatment of chronic osteoarthritis pain (lack of evidence of efficacy). | - | Use of “Versatis Medicated Plasters” in those with a record of osteoarthritis at Wave 4 | All participants with a record of osteoarthritis at Wave 4 |
| 5. Gabapentinoids (e.g., gabapentin, pregabalin) for non-neuropathic pain (lack of evidence of efficacy). | Insufficient data | - | - |
| 6. Paracetamol at doses ≥ 3 g/24 hours in patients with poor nutritional status i.e., BMI < 18 or chronic liver disease (risk of hepatotoxicity). | Insufficient data | - | - |
| **Section M: Antimuscarinic/Anticholinergic Drug Burden** |  |  |  |
| 1. Concomitant use of two or more drugs with antimuscarinic/anticholinergic properties (e.g., bladder antispasmodics, intestinal antispasmodics, tricyclic antidepressants, first generation antihistamines, antipsychotics) (risk of increased antimuscarinic/anticholinergic toxicity). | - | Use of two or more medications with anticholinergic properties: bladder antispasmodics (G04BD02, G04BD04, G04BD06, G04BD07, G04BD08, G04BD09, G04BD10, G04BD11); intestinal antispasmodics (dicycloverine, hyoscine butylbromide, propantheline bromide A03AA07; A03BB01; A03AB05); triyclic antidepressants (N06AA); first generation antihistamines (R06AA or R06AB or R06AC or RO6AD); antipsychotics (N05A) at Wave 4 | All participants with a record of using an anticholinergic medication at Wave 4 |

**Table S3. Specification of START applied to TILDA Wave 4 CAPI data**

| **Indicator** | **Reason for exclusion** | **Data operationalisation** | **Drug/disease prevalence denominator** |
| --- | --- | --- | --- |
| **Section A: Indicated drugs** |  |  |  |
| 1. Where a drug is clearly indicated and considered appropriate in the particular clinical context and there is no clear contraindication, that drug should be initiated as per formulary guidelines for dose and duration. | Insufficient data | - | - |
| **Section B: Cardiovascular System** |  |  |  |
| 1. Antihypertensive therapy where systolic blood pressure > 140 mmHg and /or diastolic blood pressure > 90 mmHg, unless established moderate or severe physical frailty in whom the threshold for therapy is 150 mmHg systolic pressure and/or 90 mmHg diastolic pressure. | Insufficient data | - | - |
| 2.  Statin therapy with a documented history of coronary, cerebral or peripheral vascular disease, unless the patient’s status is end-of-life or established moderate or severe frailty. | **-** | No use of a statin (ATC group C10AA), in those with a history of heart attack, angina, heart failure, atrial fibrillation, stroke or transient ischaemic attack at any prior wave, and without frailty at Wave 4 (as assessed using the FRAIL scale) | All participants with a record of coronary or cerebrovascular disease and any prior wave and with no record of frailty at Wave 4 |
| 3.  Angiotensin Converting Enzyme (ACE) inhibitor with coronary artery disease. | **-** | No use of ACE inhibitor (ATC group C09) in those with a record of heart attack, angina, heart failure or atrial fibrillation at any prior wave | All participants with a record of heart attack, angina, heart failure or atrial fibrillation at any prior wave |
| 4.  Beta-blocker with symptomatic coronary artery disease. | Insufficient data | - | - |
| 5.  Angiotensin Converting Enzyme (ACE) inhibitor for heart failure with reduced ejection fraction. | Insufficient data | - | - |
| 6. Cardio-selective beta-blocker (bisoprolol, nebivolol, metoprolol or carvedilol) for stable heart failure with reduced ejection fraction. | Insufficient data | - | - |
| 7.  Mineralocorticoid receptor antagonist (spironolactone, eplerenone) in heart failure without severe renal function impairment i.e., eGFR > 30 ml/min/m^2^. | Insufficient data | - | - |
| 8. SGLT-2 inhibitors (canagliflozin, dapagliflozin, empagliflozin, ertugliflozin) in symptomatic heart failure with or without reduced ejection fraction regardless of diabetes being present or not. | Insufficient data | - | - |
| 9. Sacubitril/valsartan in heart failure with reduced ejection fraction causing persistent heart failure symptoms despite optimal dose of ACE inhibitor or Angiotensin Receptor Blocker (Sacubitril/valsartan to replace ACE inhibitor or Angiotensin Receptor Blocker). | Insufficient data | - | - |
| 10. Beta-blocker for chronic atrial fibrillation with uncontrolled heart rate. | Insufficient data | - | - |
| 11.  Intravenous iron for symptomatic heart failure with reduced ejection fraction and iron deficiency. | Insufficient data | - | - |
| **Section C: Coagulation System** |  |  |  |
| 1. Vitamin K antagonists or direct thrombin inhibitors or factor Xa inhibitors in the presence of chronic or paroxysmal atrial fibrillation. | **-** | No use of ATC vitamin k antagonists (ATC group B01AA), direct thrombin inhibitors (ATC group B01AE), or factor Xa inhibitors (ATC group B01AF) in those with a record of atrial fibrillation at Wave 4 | All participants with a record of atrial fibrillation at Wave 4 |
| 2. Antiplatelet therapy (aspirin or clopidogrel or prasugrel or ticagrelor) with a documented history of coronary, cerebral or peripheral vascular disease. | **-** | No use of ATC group B01AC at Wave 4, in those with a record of heart attack, angina, heart failure, atrial fibrillation, stroke or transient ischaemic attack at any prior wave, and not receiving vitamin K antagonist, direct thrombin inhibitors or factor Xa inhibitors | All participants with a record of heart attack, angina, heart failure, atrial fibrillation, stroke or transient ischaemic attack at any prior wave |
| **Section D: Central Nervous System** |  |  |  |
| 1.  L-DOPA or a dopamine agonist in idiopathic Parkinson’s disease with functional impairment and resultant disability. | **-** | No use of ATC groups N04BA, N04BB, or N04BC, in those with a record of Parkinson’s disease, and with any impairment in activities of daily living (ADL) or instrumental activities of daily living (IADL) at Wave 4 | All participants with a record of Parkinson’s disease, and with any impairment in ADLs or IADLs at Wave 4 |
| 2.  Non-TCA antidepressant for major depression. | **-** | No use of a non-TCA antidepressant (ATC groups N06AB, N06AF, N06AG, or N06AX), or a TCA antidepressant (ATC group N06AA), in those with a CESD-8 score of 9 or more at Wave 4 | All participants with a CESD-8 score of 9 or more at Wave 4 |
| 3.  Acetylcholinesterase inhibitor (donepezil, rivastigmine, galantamine) for mild-moderate Alzheimer’s dementia. | Insufficient data | **-** | **-** |
| 4.  Rivastigmine for Dementia with Lewy Bodies or Parkinson’s disease dementia. | Insufficient data | **-** | **-** |
| 5.  Selective serotonin reuptake inhibitor (or SNRI or pregabalin if SSRI contraindicated) for persistent severe anxiety that affects independent functioning and quality of life. | Insufficient data | **-** | **-** |
| 6.  Dopamine agonist (ropinirole or pramipexole or rotigotine) for Restless Legs Syndrome, once iron deficiency and severe chronic kidney disease (i.e., eGFR < 30 ml/min/m^2^) have been excluded. | Insufficient data | **-** | **-** |
| 7.  Propranolol for essential tremor with functional impairment and resultant disability. | Insufficient data | **-** | **-** |
| **Section E: Renal System** |  |  |  |
| 1. One-alpha hydroxycholecalciferol or calcitriol supplementation in severe chronic kidney (i.e., eGFR < 30 ml/min/m^2^) disease with hypocalcaemia (corrected serum calcium < 2.10 mmol/l) and associated secondary hyperparathyroidism. | Insufficient data | **-** | **-** |
| 2. Phosphate binder in severe chronic kidney disease (i.e., eGFR < 30 ml/min/m2) if serum phosphate concentration persistently >1.76 mmol/l (5.5 mg/dl) despite adherence to renal diet. | Insufficient data | **-** | **-** |
| 3. Erythopoietin analogue in severe chronic kidney disease (i.e., eGFR < 30 ml/min/m^2^) with symptomatic anaemia not attributable to haematinic or iron deficiency to achieve a haemoglobin concentration of 10.0 to 12.0 g/dl. | Insufficient data | **-** | **-** |
| 4.  Angiotensin receptor blocker (ARB) or Angiotensin Converting Enzyme Inhibitor (ACE-I) in chronic kidney disease with proteinuria i.e., urine albumin excretion >300 mg/24 hours. | Insufficient data | **-** | **-** |
| **Section F: Gastrointestinal System** |  |  |  |
| 1.  Proton Pump Inhibitor with severe gastro-oesophageal reflux disease or peptic oesophageal stricture requiring dilatation. | Insufficient data | **-** | **-** |
| 2.  Proton pump inhibitor with initiation of low-dose aspirin and previous history of peptic ulcer or reflux oesophagitis. | **-** | Use of aspirin at Wave 4 assumed to be use of low-dose aspirin when ATC code B01AC06 used. No use of a proton pump inhibitor (ATC group A02BC), in those with ATC code B01AC06, and with a record of ulcer at any prior wave | All participants with use of B01AC06 and with a record of ulcer at any prior wave |
| 3.  Proton pump inhibitor with short-term (< 2 weeks) or longer-term (> 2 weeks) NSAID. | **-** | Use of NSAID (M01AE, M01AC, M01AG or M01AB) at Wave 4 only assumed to be short term use, with use at Wave 3 and Wave 4 assumed to be long term use. No use of a proton pump inhibitor (ATC group A02BC) in those with use of NSAID at Wave 4 | All participants with use of NSAID (M01AE, M01AC, M01AG or M01AB) at Wave 4 |
| 4.  Fibre supplements (e.g., bran, ispaghula, methylcellulose, sterculia) for diverticulosis with a history of constipation. | Insufficient data | **-** | **-** |
| 5.  Osmotic laxative (e.g., lactulose, macrogol, sorbitol) for chronic persistent idiopathic or secondary benign constipation. | Insufficient data | **-** | **-** |
| 6.  Probiotics used with antibiotics in patients who are not immunocompromised or severely debilitated for the prevention of Clostridioides difficile-associated diarrhoea. | Insufficient data | **-** | **-** |
| 7.  Helicobacter pylori eradication therapy in HP-associated active peptic ulcer disease. | Insufficient data | **-** | **-** |
| **Section G: Respiratory System** |  |  |  |
| 1. Long-acting muscarinic antagonist (LAMA e.g., tiotropium, aclidinium, umeclidinium, glycopyrronium) or long-acting beta 2 agonist (LABA e.g., bambuterol, formoterol, indacaterol, olodaterol, salmeterol) for symptomatic COPD of GOLD 1 or 2 severity and chronic asthma. | Insufficient data | **-** | **-** |
| 2. Regular i.e. daily inhaled corticosteroid (e.g., beclomethasone, budesonide, ciclesonide, fluticasone, mometasone) for moderate-severe asthma or COPD of GOLD 3 or 4 severity, where FEV1 <50% of predicted value and repeated exacerbations requiring treatment with oral corticosteroids. | Insufficient data | **-** | **-** |
| 3. Home continuous oxygen with documented chronic hypoxaemia (i.e., pO2 < 8.0 kPa or 60 mmHg or SaO2 < 89%). | Insufficient data | **-** | **-** |
| **Section H: Musculoskeletal System** |  |  |  |
| 1.  Disease-modifying anti-rheumatic drug (DMARD) with chronic, active and disabling rheumatoid arthritis. | **-** | No use of a DMARD (ATC L01BA01, L04AA24, L01FA01, L04AC03, or P01BA02) in those with a record of rheumatoid arthritis, and with any ADL or IADL impairment at Wave 4 | All participants with a record of rheumatoid arthritis and with any ADL or IADL impairment at Wave 4 |
| 2.  Bisphosphonates and vitamin D and calcium in patients taking long-term systemic corticosteroid therapy for prevention of steroid-induced osteoporosis. | **-** | Use of systemic corticosteroids (ATC groups H02AA or H02AB) at Wave 3 and Wave 4 assumed continuous use. No use of a bisphosphonate (ATC group M05BA) and no use of calcium and vitamin D (ATC group A12AX) in those with use of systemic corticosteroids at Wave 3 and Wave 4. | All participants with a record of systemic corticosteroid use at both Wave 3 and Wave 4 |
| 3.  Vitamin D in patients with known osteoporosis and/or previous fragility fracture(s) and/or Bone Mineral Density T-scores below -2.5 in one or multiple sites. | **-** | No use of vitamin D (ATC A11CC) or calcium and vitamin D (ATC A12AX) in those with a record of osteoporosis at Wave 4 | All participants with a record of osteoporosis at Wave 4 |
| 4.  Bone anti-resorptive or anabolic therapy (e.g., bisphosphonate, teriparatide, denosumab) in patients with documented osteoporosis (Bone Mineral Density T-scores below -2.5 in one or multiple sites) and/or previous history of fragility fracture(s) – where no pharmacological or clinical status contraindication exists such as poor one-year life expectancy. | Insufficient data | **-** | **-** |
| 5.  Vitamin D supplement in older people with confirmed 25-hydroxycolecalciferol deficiency (< 20 micrograms/L, < 50 nmol/L) who are housebound or experiencing falls or with osteopenia (Bone Mineral Density T-score is less than -1.0 but above -2.5 in one or multiple sites). | Insufficient data | **-** |  |
| 6.  Anti-resorptive treatment after discontinuation of at least two doses of denosumab (rebound increased bone turnover markers, BMD loss, and increased risk of vertebral fracture following denosumab discontinuation). | Insufficient data | **-** | **-** |
| 7.  Anti-resorptive treatment after discontinuation of teriparatide/abaloparatide treatment for osteoporosis. | **-** | No use of antiresorptive treatment (M05BA or M05BX04) in those who had use of teriparatide or abaloparatide (H05AA02, H05AA04) at Wave 3 but not at Wave 4 (assumed discontinuation), and with a record of osteoporosis at Wave 3 and Wave 4 | All participants with use of teriparatide/abaloparatide at Wave 3 but not at Wave 4, and with a record of osteoporosis at Wave 3 and Wave 4 |
| 8.  Xanthine-oxidase inhibitors (e.g. allopurinol, febuxostat) with a history of recurrent episodes of gout. | **-** | No use of xanthine-oxidase inhibitors (ATC group M04AA) with recurrent episodes of code, defined as the use of anti-gout medications (ATC group M04A) at waves 1, 2 and 3 | All participants with use of anti-gout medications (ATC group M04A) at waves 1, 2 and 3 |
| 9.  Folic acid supplement in patients taking methotrexate. | **-** | No use of folic acid (ATC code B03BB01) in those with methotrexate (ATC code L01BA01) use at Wave 4 | All participants with use of methotrexate at Wave 4 |
| **Section I: Urogenital System** |  |  |  |
| 1.  Selective alpha-1 receptor blocker (e.g., tamsulosin, silodosin) for lower urinary tract symptoms related to benign prostatic hyperplasia where prostatectomy is not considered necessary or appropriate or safe. | Insufficient data | - | - |
| 2.  5-alpha reductase inhibitor (e.g., finasteride, dutasteride) for lower urinary tract symptoms related to benign prostatic hyperplasia where prostatectomy is not considered necessary or appropriate or safe. | Insufficient data | - | - |
| 3.  Topical vaginal oestrogen or vaginal oestrogen pessary for symptomatic atrophic vaginitis. | Insufficient data | - | - |
| 4.  Topical vaginal oestrogen or vaginal oestrogen pessary in women for recurrent urinary tract infections. | Insufficient data | - | - |
| 5.  Phosphodiesterase type-5 inhibitors (e.g., avanafil, sildenafil, tadalafil, vardenafil) for persistent erectile dysfunction that causes distress. | Insufficient data | - | - |
| **Section J: Endocrine System** |  |  |  |
| 1. ACE inhibitor or Angiotensin Receptor Blocker (if intolerant of ACE inhibitor) in diabetes with evidence of renal disease i.e., dipstick proteinuria or microalbuminuria (>30 mg/24 hours) unless evidence of severe CKD (eGFR < 30 ml/min/m^2^). | Insufficient data | **-** | **-** |
| **Section K: Analgesics** |  |  |  |
| 1.  High-potency opioids in moderate-severe non-arthritis pain, where paracetamol, NSAIDs or low-potency opioids are not appropriate to the pain severity or have been ineffective. | Insufficient data | **-** | **-** |
| 2.  Laxatives in patients receiving opioids regularly i.e., other than PRN use. | **-** | Use of opioids (ATC group N02A) at Wave 3 and Wave 4 assumed to be regular use. No use of laxatives (ATC group A06A) in those with opioid use at Wave 3 and Wave 4 | All participants with use of opioids (N02A) at both Wave 3 and Wave 4 |
| 3.  Topical 5% lidocaine (lignocaine) patch for localized neuropathic pain, e.g. post-herpetic neuralgia. | Insufficient data | **-** | **-** |
| **Section L: Vaccines** |  |  |  |
| 1.  Seasonal influenza vaccine annually. | **-** | No receipt of winter flu vaccine in both winter 2015/16 and 2016/17 seasons | **-** |
| 2.  Pneumococcal vaccine at least once according to national guidelines. | Insufficient data | **-** | **-** |
| 3.  Varicella-zoster vaccine according to national guidelines. | Insufficient data | **-** | **-** |
| 4.  SARS-CoV2 vaccine according to national guidelines. | Non-applicable | **-** | **-** |
