# Supplementary material for: Prevalence of potentially inappropriate prescribing in community-dwelling older adults: an application of STOPP/START version 3 to The Irish Longitudinal Study on Ageing (TILDA)
Source: Eur Geriatr Med. 2025 Apr 28;16(4):1389–402. doi: 10.1007/s41999-025-01201-3 (PMC12378767; doi:10.1007/s41999-025-01201-3)
Supplement: Supplementary file 3 — Supplementary file3 (DOCX 22 KB) [file 41999_2025_1201_MOESM3_ESM.docx]

**ADDITIONAL FILE 3**

**Table S4. Chronic conditions included in the definition of multimorbidity (based upon Larkin et al 2022)[1]**

| **Chronic condition** | **Specific diagnoses included** |
| --- | --- |
| Cardiovascular disease (any) | Heart attack, or angina, or heart rhythm disorder or heart failure or atrial fibrillation |
| Cerebrovascular disease (any) | Stroke or transient ischaemic attack |
| Hypertension | Hypertension |
| Respiratory disease | Asthma or chronic lung disease such as chronic bronchitis or emphysema |
| Diabetes (any) | Type 1 or Type 2 diabetes |
| Hypercholesterolemia | Hypercholesteremia |
| Liver disease | Alcohol abuse, liver disease or cirrhosis |
| Eye disease | Cataracts, glaucoma or age-related macular degeneration |
| Cognitive impairment | Alzheimer’s disease, dementia or other serious cognitive impairment |
| Arthritis | Osteoarthritis or rheumatoid arthritis |
| Osteoporosis | Osteoporosis |
| Cancer | Cancer or a malignant tumour |
| Parkinson’s disease | Parkinson’s disease |
| Emotional/ psychological conditions | Anxiety, depression, manic depression, schizophrenia, psychosis or hallucinations |
| Substance abuse | Substance abuse |
| Stomach ulcers | Ulcers |
| Varicose ulcers | Varicose ulcers |
| Thyroid disease | Thyroid problems |
| Anaemia | Severe anaemia |
| Chronic kidney disease | Chronic kidney disease |
| Epilepsy | Epilepsy |

**References**

[1] Larkin J, Walsh B, Moriarty F, Clyne B, Harrington P, Smith SM. What is the impact of multimorbidity on out-of-pocket healthcare expenditure among community-dwelling older adults in Ireland? A cross-sectional study. BMJ Open 2022;12:e060502. https://doi.org/10.1136/bmjopen-2021-060502.
