# Supplementary material for: Prevalence of potentially inappropriate prescribing in community-dwelling older adults: an application of STOPP/START version 3 to The Irish Longitudinal Study on Ageing (TILDA)
Source: Eur Geriatr Med. 2025 Apr 28;16(4):1389–402. doi: 10.1007/s41999-025-01201-3 (PMC12378767; doi:10.1007/s41999-025-01201-3)
Supplement: Supplementary file 4 — Supplementary file4 (DOCX 20 KB) [file 41999_2025_1201_MOESM4_ESM.docx]

**ADDITIONAL FILE 4**

**Table S5. START Version 3 criteria applied to TILDA data for all those aged ≥65 years at Wave 4 (N=3,619)**

| **START criteria description** | **PPO (n)** | **PPO (%)** | **Prescribing per indication^a^ (%)** |
| --- | --- | --- | --- |
| ***Cardiovascular system*** |  |  |  |
| Statin therapy with a documented history of coronary, cerebral or peripheral vascular disease and no frailty | 341 | 9.42 | 38.27 |
| Angiotensin Converting Enzyme (ACE) inhibitor with coronary artery disease | 601 | 16.61 | 74.11 |
| ***Coagulation system*** |  |  |  |
| Vitamin K antagonists or direct thrombin inhibitors or factor Xa inhibitors in the presence of atrial fibrillation | 80 | 2.21 | 51.95 |
| Antiplatelet therapy with a documented history of coronary, cerebral or peripheral vascular disease | 282 | 7.79 | 28.78 |
| ***Central nervous system*** |  |  |  |
| L-DOPA or a dopamine agonist in Parkinson’s disease with functional impairment and resultant disability | 15 | 0.41 | 55.56 |
| Non-TCA antidepressant for major depression | 267 | 7.38 | 76.95 |
| ***Gastrointestinal system*** |  |  |  |
| Proton pump inhibitor with aspirin and previous history of peptic ulcer | 35 | 0.97 | 40.70 |
| Proton pump inhibitor with short-term (< 2 weeks) or longer-term (> 2 weeks) NSAID | 100 | 2.76 | 68.03 |
| ***Musculoskeletal system*** |  |  |  |
| Disease-modifying anti-rheumatic drug (DMARD) with chronic, active and disabling rheumatoid arthritis | 76 | 2.10 | 97.44 |
| Bisphosphonates and vitamin D and calcium in patients taking long-term systemic corticosteroid therapy | 26 | 0.72 | 63.41 |
| Vitamin D in patients with known osteoporosis | 439 | 12.13 | 64.46 |
| Anti-resorptive treatment after discontinuation of teriparatide/abaloparatide treatment for osteoporosis | 4 | 0.11 | 100 |
| Xanthine-oxidase inhibitors with a history of recurrent episodes of gout | 4 | 0.11 | 9.30 |
| Folic acid supplement in patients taking methotrexate | 5 | 0.14 | 23.81 |
| ***Analgesics*** |  |  |  |
| Laxatives in patients receiving opioids regularly | 74 | 2.04 | 90.24 |
| ***Vaccinations*** |  |  |  |
| Seasonal influenza vaccine annually | 47 | 1.30 | **-** |
|  |  |  |  |
| **Any START indicator** | 1,309 | 36.17 | **-** |
| ***1 indicator*** | 788 | 21.77 | **-** |
| ***≥ 2 indicators*** | 521 | 14.40 | **-** |

***^a^*** *Calculated as the proportion of overall disease or medication prevalence (e.g. use of medication with potent anticholinergic/antimuscarinic effects in participants with dementia as a proportion of dementia prevalence). Further detail on the disease/medication denominator used for each criterion is provided in Additional File 2.*
